# Disarming *Staphylococcus aureus* from destroying human cells by simultaneously neutralizing six cytotoxins with two human monoclonal antibodies

**DOI:** 10.1080/21505594.2017.1391447

**Published:** 2017-12-26

**Authors:** Harald Rouha, Susanne Weber, Philipp Janesch, Barbara Maierhofer, Karin Gross, Ivana Dolezilkova, Irina Mirkina, Zehra C. Visram, Stefan Malafa, Lukas Stulik, Adriana Badarau, Eszter Nagy

**Affiliations:** Arsanis Biosciences, Helmut-Qualtinger-Gasse 2, Campus Vienna Biocenter, Vienna, Austria

**Keywords:** *Staphylococcus aureus*, leukocidins, alpha-hemolysin, toxin neutralization, human monoclonal antibodies, host defense

## Abstract

Pathogenesis of *Staphylococcus aureus* is increasingly recognized to be driven by powerful toxins. *Staphylococcus aureus* employs up to six pore-forming toxins to subvert the human host defense and to promote bacterial invasion: alpha-hemolysin that disrupts epithelial and endothelial barriers and five leukocidins that lyse phagocytes involved in bacterial clearance. Previously, we described two human monoclonal antibodies (mAbs), ASN-1 that neutralizes alpha-hemolysin and four leukocidins (LukSF-PV, LukED, HlgAB, HlgCB), and ASN-2 that inactivates the 5^th^ leukocidin, LukGH. In this study we tested the individual and combined effects of ASN-1 and ASN-2 in multiple *in vitro* models employing relevant human target cells. We found that diverse *S. aureus* isolates with different genetic backgrounds (based on MLST- and *spa*-typing) and antibiotic sensitivity (both MRSA and MSSA) displayed greatly different cytotoxin expression patterns influenced by the type of growth medium used. Both mAbs were required to fully prevent the lysis of human neutrophils exposed to the mixture of recombinant cytotoxins or native toxins present in the culture supernatants of *S.* *aureus* isolates. Flow cytometry confirmed the protective effects of ASN-1 + ASN-2 (known as ASN100) on granulocytes, monocytes, NK-cells and T-lymphocytes. ASN-1 alone preserved the integrity of a 3D-primary culture of human tracheal/bronchial mucociliary epithelial tissue infected with *S. aureus*. We conclude that simultaneous inhibition of alpha-hemolysin and five leukocidins by ASN100 blocks cytolytic activity of *S. aureus* towards human target cells *in vitro*.

## Introduction

*Staphylococcus aureus* is a major human pathogen associated with a significant global health care burden. Approximately 30% of the human population is colonized. However, *S. aureus* can change from a commensal to a pathogen, causing severe disease, such as pneumonia, blood stream infections, osteomyelitis, and complicated skin and deep tissue infections.^1,2^ Methicillin-resistant *S. aureus* (MRSA) has become a global problem and is responsible for life-threatening infections even in young and healthy individuals outside of hospital settings. Despite the availability of appropriate antibiotics, severe MRSA and MSSA infections remain associated with high mortality.^1^

The primary innate defense mechanism against *S. aureus* is the control of bacterial growth by phagocytic uptake and bacterial killing, predominantly by neutrophilic granulocytes.^3^ Antibodies binding to the bacterial surface and activating the complement system greatly enhance this process (opsonization). Frequent exposure to *S. aureus* results in increasing anti-staphylococcal antibody titers during childhood, and human sera exhibit opsonophagocytic activity *in vitro*.^4^ Despite this, *S. aureus* infections occur repeatedly. All anti-staphylococcal vaccine and passive immunization approaches tested to date in pivotal clinical trials have failed. They have all targeted a single surface structure (i.e. protein, carbohydrate capsule, or LTA) and aimed at opsonophagocytic killing (OPK) as the mode of action. The supportive pre-clinical data, including efficacy in rodent models and *in vitro* OPK activity, generated with these product candidates raises concerns about the relevance of the animal models applied and OPK as a main mechanism of anti-*S. aureus* antibodies.^5,6^

One of the explanations for the lack of efficacy of antibodies binding to the bacterial surface is the numerous secreted toxins that are produced by *S. aureus* to target host cells for counteracting phagocytosis and to disrupt tissue integrity. Uniquely among bacterial pathogens, *S. aureus* secretes up to six different beta-barrel pore forming cytotoxins.^7-9^ Alpha-hemolysin (Hla) is the best characterized *S. aureus* virulence factor with proven contribution to pathogenesis in all animal models tested.^10^ It lyses epithelial and endothelial cells, and is also toxic to lymphocytes and monocytes.^10^ The other five pore forming cytotoxins – Leukocidin SF-PV (Panton-Valentine leukocidin), ED and GH (the latter also called LukAB) and the two gamma-hemolysins (HlgAB and HlgCB) – all target leukocytes, and primarily attack phagocytic cells, such as granulocytes, macrophages, and monocytes.^11^ In addition, LukED lyses lymphocytes and displays hemolytic activity similar to the gamma-hemolysins.^8,9,11^ Hla and LukGH have also been shown to contribute to biofilm production based on gene deletion strains and studies with neutralizing antibodies.^12,13^

The *S. aureus* leukocidins are highly adapted to the human host and do not elicit appreciable toxicity towards rodent cells with the exception of LukED.^14,15^ Since mice and rats are the most commonly used species for *S. aureus* disease models, the important role of the leukocidins in pathogenesis has been recognized only recently. The lack of phenotype of *lukSF-PV* deletion mutant strains of *S. aureus* in mouse and non-human primate models is explained now by the resistance of phagocytes of these species towards LukSF-PV.^16^ Since rabbit phagocytic cells are susceptible to the cytotoxic effect of all *S. aureus* leukocidins, the rabbit represents a more relevant species than the mouse. The prominent role of LukSF-PV in pneumonia pathogenesis was proven in rabbits.^17^ Based on *in vitro* assays, the sensitivity of rabbit PMNs for LukSF-PV and gamma hemolysin is comparable to that of human neutrophils, however, LukED is approximately 100-fold more potent and LukGH is 100-fold less toxic to rabbits cells.^14,15,18^

Species specificity of the *S. aureus* cytotoxins became understood at the molecular level following the identification of their cellular receptors that all belong to chemokine (CXCR1, CXCR2, CCR2, CCR5, DARC) and complement receptor families (C5aR, C3R/CD11b).^11,19–24^ These receptors are either not expressed or not sufficiently conserved in rodents. ADAM10, the receptor for Hla is expressed on human and animal epithelial and endothelial cells, as well as on red blood cells of species susceptible to hemolysis by Hla.^25^

Dissecting the role of these cytotoxins in human pathogenesis has begun, but as there are little data regarding *in vivo* expression and correlation with disease severity, more efforts and complex approaches are needed. The relevance of LukSF-PV was initially indicated by gene prevalence studies demonstrating the presence of *lukSF-PV* in highly successful CA-MRSA strains causing severe *S. aureus* infections, such as necrotizing pneumonia and community associated MRSA infections in otherwise healthy people.^26,27^ These observations triggered interest in the other leukocidins. Since the genes encoding Hla, HlgAB, HlgCB, and LukGH are part of the core genome of *S. aureus*, and *lukED* is present in more than 50% of strains, gene prevalence studies provide limited information regarding their role in pathogenesis. Hla expression levels of MSSA isolates have been shown to be associated with the ability to cause pneumonia in mechanically ventilated patients.^28^ Anti-Hla antibodies were shown to be protective in mouse models of pneumonia, bacteremia, and skin infections,^29-31^ and suggested to be protective in humans as well based on the inverse correlation between serum levels and severe *S. aureus* disease in children and adults.^32,33^ It seems that natural infection in general does not provoke durable immunity, since patients can experience recurrent *S. aureus* infections.^6^ Nevertheless, anti-Hla and anti-LukSF-PV antibody levels were shown to inversely correlate with severity in necrotizing pneumonia.^33,34^

Stimulated by the mounting evidence supporting the important role of the leukocidins in *S. aureus* infections, we have developed human monoclonal antibodies (mAbs) to neutralize Hla and the five leukocidins. A unique type of cytotoxin cross-reactive antibodies that inactivate four of the five leukocidins, HlgAB, HlgCB, LukED and LukSF-PV, and Hla was discovered and one of these mAbs, Hla-F#5 was selected as a development lead, and termed ASN-1.^35^ ASN-1 recognizes a conserved structural epitope in Hla, HlgB, LukF-PV, and LukD with high affinity.^35^ ASN-1 was shown to be fully protective in a lethal CA-MRSA USA300 necrotizing pneumonia model in rabbits, while monospecific Hla mAbs tested in the same model were only partially efficacious.^15,36^ Since the binding epitope of ASN-1 is not conserved in LukGH, an additional mAb, targeting this toxin was also developed. Based on its high binding affinity and potent neutralization of the different sequence variants of LukGH, the αLukGH-mAb#5.H1H2 was selected as development lead, named ASN-2.^37^ The combination of ASN-1 and ASN-2, called ASN100, is currently being tested in a Phase 2 clinical trial for the prevention of *S. aureus* pneumonia in mechanically ventilated patients (NCT02940626).

Due to species specificity, *in vitro* model systems employing human target cells are necessary to assess the individual and collective contribution of the leukocidins to phagocyte damage. In this study, we applied a comprehensive approach using multiple isolates and different culture conditions to gain insights into the cytotoxin expression profiles of *S. aureus* and to evaluate the protective effects of the two cytotoxin neutralizing mAbs, ASN-1 and ASN-2, on human cells.

## Methods

### Bacterial strains, culture conditions and preparation of culture supernatants

The USA300 CA-MRSA strain TCH1516 was obtained from ATCC (ATCC® BAA-1717™). Isogenic mutants lacking the *hla, hlgABC, lukED, lukSF-PV* and *lukGH* genes were generated in the TCH1516 background by homologous recombination as described previously.^35^ Wild-type and mutant strains showed comparable growth characteristics based on OD_600 nm_ measurements and CFU counting on sheep blood agar plates.^38^ 18 additional *S. aureus* strains used in this study are listed in Table 1.

**Table 1.** *S. aureus* strains used in the study.

	MLST-*SCCmec*-*spa*-type or clonal complex	*MRSA/MSSA*	*lukSF-PV*	*lukED*	*hlgABC*	*lukGH*	*hla*	Source of strain
**Prototype wild-type strains**								
TCH1516	ST8-IV-t622	MRSA	+	+	+	+	+	ATCC BAA-1717™
USA300 CA-MRSA								
NRS382	ST5-II-t002	MRSA	−	+	+	+	+	NARSA^a^
USA100 HA-MRSA								
MRSA252	ST36-II-t018	MRSA	−	+	+	+	– ^**^	ATCC BAA-1720™
USA200 European HA-MRSA								
NRS385	ST8-IV-t064	MRSA	−	+	+	+	+	NARSA^a^
USA500 HA-MRSA								
NRS386	ST72-IVa-t126	MRSA	+	+	+	+	+	NARSA^a^
USA700 CA-MRSA								
MW2	ST1-IV-t125	MRSA	+	+	+	+	+	ATCC BAA-1707™
USA400								
Newman	ST254-t008	MSSA	−	+	+	+	+	Prof. Claire Poyart, Hôpital Cochin, Paris, France
H19Live-stock MRSA	CC10	MRSA	−	+	+	+	+	Prof. Ashley Robinson, University of Mississippi Medical Center, USA

**Gene deletion mutant strains**^b^								
TCH1516 Δ*hla/hlgABC/lukSF/lukED/lukGH* or Δall toxin mutant	ST8-IV-t622	MRSA	−	−	−	−	−	In house^b^
TCH1516Δ*lukGH*	ST8-IV-t622	MRSA	+	+	+	−	+	In house^b^
TCH1516Δ*hla/hlgABC/lukSF/lukED* or *lukGH* only	ST8-IV-t622	MRSA	−	−	−	+	−	In house^b^
TCH1516Δ*hla*	ST8-IV-t622	MRSA	+	+	+	+	−	In house^b^
TCH1516Δ*hlgABC/lukSF/lukED/lukGH* or *hla* only	ST8-IV-t622	MRSA	−	−	−	−	+	In house^b^
**Human clinical isolates**^c^								
LA#3	ST1970-t065	MSSA	−	−	+	+	+	Lahey Medical Center^c^
LA#5	ST8-IV-t008	MRSA	−	+	+	+	+	Lahey Medical Center^c^
LA#16	ST81-t127	MSSA	−	+	+	+	+	Lahey Medical Center^c^
LA#45	ST121-t645	MSSA	−	+	+	+	+	Lahey Medical Center^c^
LA#48	ST45-t040	MSSA	−	−	+	+	+	Lahey Medical Center^c^
LA#66	ST5-II-t002	MRSA	−	+	+	+	+	Lahey Medical Center^c^
LA#132	ST72-t148	MSSA	−	+	+	+	+	Lahey Medical Center^c^
LA#164	ST188-t189	MSSA	−	+	+	+	+	Lahey Medical Center^c^
LA#178	ST8-t334	MSSA	−	+	+	+	+	Lahey Medical Center^c^
LA#186	ST5-II-t002	MRSA	−	+	+	+	+	Lahey Medical Center^c^
LA#217	ST8-IV-t008	MRSA	+	+	+	+	+	Lahey Medical Center^c^

Notes.

** mutated (early STOP codon).

a Isolate was obtained through the Network on Antimicrobial Resistance in Staphylococcus Aureus (NARSA) program supported under NIAID/NIH contract HHSN272200700055C.

b *S. aureus* isogenic mutant strain generated in house based on the TCH1516 BAA-1717™ background.

c *S. aureus* isolates collected from ETA of ventilated patients. Stulik et al. AJRCCM, 2014.^28^

For bacterial culture supernatants (CS) generated in CCY (3% wt/vol yeast extract, 2% Bacto-Casamino acids, 2.3% sodium pyruvate, 0.63% Na_2_HPO_4_, and 0.041% KH_2_PO_4_), BHI (Fluka), TSB (Corning) and RPMI-CAS (RPMI-1640 Medium, Gibco; 1% Casamino acids, Amresco), overnight cultures obtained from a single colony were diluted to OD_600 nm_: 0.03 in fresh culture medium and grown to stationary phase for 8 hours. All tested *S. aureus* strains reached similar bacterial densities within a given medium. CS were harvested by culture centrifugation at 5000 x g at 4°C, followed by filter sterilization using 0.1 µm pore size PVDF syringe filters (Millipore).

### Monoclonal antibodies

ASN-1 and ASN-2 were produced at Boehringer Ingelheim in stably transformed CHO cell lines. Monospecific antibodies against Hla, HlgB, and LukD were derived from the yeast expressed human IgG1 libraries selected with the respective recombinant monomers, as reported previously.^35^ Human isotype control mAb (Motavizumab) and monospecific mAbs used for immunoblotting were produced in CHO-3E7 cells transiently transfected with mammalian expression plasmid pTT5 (National Research Council, Canada) encoding human antibody (IgG1) heavy and light chains using PEI MAX™ transfection reagent (Polysciences). IgGs were purified by Protein A or Protein G affinity chromatography and purities and monomeric states determined by SDS-PAGE and SEC, respectively, were both above 95%.

### Recombinant toxins

*S. aureus* leukocidins were expressed based on the USA300 CA-MRSA genome sequences (TCH1516 strain), for LukGH also additional variants derived from MRSA252, H19 and MSHR1132 were generated, as described previously.^38,39^ Hla was expressed without tag and purified by cation exchange and size exclusion chromatography. Purity was controlled by SDS-PAGE.

### Purification of peripheral white blood cells (WBCs) and PMNs

Human peripheral WBCs were isolated from heparinized blood using HetaSep (StemCell Technologies) gravity sedimentation, platelet removal by centrifugation and lysis of remaining erythrocytes using hypotonic sodium chloride solution (0.2%).

PMNs were isolated using Percoll Plus (GE Healthcare) gradient centrifugation, as described previously.^37^ PMN purification resulted in cell purity of >95% determined microscopically by Giemsa staining as well as by flow cytometric analysis of forward- versus side-scatter properties. Cell viability was >98% for PMNs and WBCs based on Trypan blue (Thermo Fisher Scientific) exclusion.

### In vitro cytotoxicity and toxin neutralization assays

PMN cytotoxicity induced by recombinant toxins and culture supernatants was measured in 96-well format as described previously.^35,38^ PMNs (2.5 × 10^4^ cells/well) were exposed to equimolar mixtures of S- and F-components for the bi-component toxins LukSF-PV, HlgAB, HlgCB, LukED or the co-expressed LukGH dimer in neutrophil medium (RPMI-1640 with 10% FCS (Sigma-Aldrich), 2 mM L-glutamine (Thermo Fisher Scientific)), for 4 hours at 37 °C, 5% CO_2_. Cell viability was determined using the Cell Titer-Glo® Luminescent Cell Viability Assay Kit (Promega) according to manufacturer's instructions with a Synergy™ HT Multi-Mode Microplate Reader (BioTek). Percent cell viability after toxin exposure was calculated relative to mock treated cells. Data were analyzed by non-linear regression using Prism 6 (Graph Pad). The toxin monomers alone did not affect PMN viability in the concentration range tested. For toxin and CS neutralization assays, mAbs and toxins/CS were pre-incubated for 30 min at room temperature (RT) prior to addition to the target cells. A human IgG1 isotype control mAb was included in all experiments.

### PMN infection assays with live bacteria

Overnight cultures of *S. aureus* grown in RPMI-CAS were diluted 1:50 and grown to mid-log phase (OD_600 nm_: 0.5) at 37°C. Bacteria were harvested, washed with PBS to remove secreted toxins, re-suspended in RPMI-1640 with 10% FCS, 2 mM L-glutamine and 10 mM HEPES (Sigma) and added to 2.5 × 10^4^ PMNs/well at different MOIs. Reactions were incubated for 90 min at 37 °C and 5% CO_2_, followed by addition of Calcein-AM fluorescent viability dye (eBioscience) at a final concentration of 4 µM for an additional 30 min. Fluorescence (λex = 485 nm, λem = 528 nm) was quantified on a Synergy™ HT Multi-Mode Microplate Reader (BioTek). Percent viability was calculated relative to mock-treated cells (100% viability).

For May-Grünwald-Giemsa staining PMNs at a concentration of 7.5 × 10^6^/mL were infected at MOI 50 in 4 mL round bottom tubes in 50 mg/mL human albumin (Biotest) in RPMI + 2 mM L-glutamine + 2 mg/mL sodium bicarbonate, pH ∼7.5 (Sigma) and incubated for 2 hours at 37 °C, 5% CO_2_. The cell suspension was then concentrated 10-fold by centrifugation at 280 x g and re-suspended in fresh medium. 10 µL of each sample were placed onto glass slides, spread and allowed to air-dry. Cell smears were stained with May-Grünwald Solution (Sigma) for 3 min and for 30 min with 10% Giemsa solution (Sigma), washed with dH_2_0 and air-dried. Images were taken with a ZEISS 100x Plan-APOCHROMAT (1.4 NA) on a ZEISS Imager M1 microscope equipped with AxioCamMRc5 color camera.

### Flow cytometry based surface staining of peripheral white blood cells

ASN100 mediated protection of human peripheral white blood cells was measured by flow cytometry in 96-well plate format. WBCs (1 × 10^6^/mL) were exposed to a pool of early stationary phase BHI cultures of TCH1516 and the six clinical isolates (indicated in Fig. 2B) in presence and absence of control antibody or toxin neutralizing mAbs. Following a 2 hour incubation at 37 °C and 5% CO_2_, cells were pelleted by centrifugation (5 min, 1000 x g, RT) and stained with fluorochrome-labelled antibodies (BioLegend if not stated otherwise) specific for CD3 (clone HIT3a, APC), CD14 (clone 63D3, PE/Cy7), CD19 (clone HIB19, Alexa Fluor488) and CD56 (eBioscience; clone TULY56, eFluor 450) and the corresponding isotype control antibodies in Hanks' Balanced Salt Solution (HBSS, Thermo Fisher Scientific) supplemented with 0.5% BSA (Biomol) and 0.01% NaN_3_ for 15 min at RT with constant mild agitation. Samples were measured with a CytoFLEX flow cytometer (Beckman Coulter) and data were analyzed with FCS Express version 5.0 (De Novo Software). Side-scatter and CD14 expression were used to discriminate lymphocyte, granulocyte and monocyte populations. Within the lymphocyte gate, T-cells, NK-cells, and B-cells were quantified based on expression of CD3, CD56, and CD19 respectively.

### Human 3D lung tissue infection model

EpiAirway™ tissues were obtained from MatTek. Upon delivery, the tissues were processed according to the supplier's protocol. For infection, PBS washed bacteria obtained from RPMI-CAS mid-log cultures were applied to the apical surface (MOI 100) in presence of either ASN-1, ASN-2, the mixture of these mAbs or an isotype control mAb (2 µM each) in 30 µL volumes. MAbs were also added to the basal feeding medium at the same concentration. Following incubation for 24 hours at 37 °C and 5% CO_2_, the basolateral medium was harvested for measuring LDH release using the CytoTox-ONE™ Homogeneous Membrane Integrity LDH release Assay (Promega) and for subsequent measurement of PMN toxicity in a Cell Titer-Glo® Luminescent Cell Viability Assay Kit (Promega) as described above. Saponin in dH_2_O (1%) was used as lysis control in LDH release assays. Bacteria on the apical surface of the tissue were collected by three washing steps with 300 µL PBS and serial dilutions were plated on sheep blood agar plates for CFU determination. The washed tissues were fixed overnight with a total of 2 mL 4% paraformaldehyde (Sigma) at 4 °C, embedded in paraffin and 1 µm sections were stained with hematoxylin and eosin at the automated slide stainer Gemini AS (Histocom). Images were taken with a ZEISS EC Plan-Neofluar 20x/0.50 objective on a ZEISS Imager M1 microscope equipped with AxioCamMRc5 color camera.

### Immunoblotting to detect cytotoxin expression

Early stationary phase CS generated in different media were separated by SDS–PAGE under reducing conditions. Proteins were then transferred to PVDF (Hla, LukG and LukS-PV) and nitrocellulose membranes (LukD, HlgB) using a Trans-Blot Turbo device (BioRad). Hla, LukD and HlgB expression was assessed using monospecific human antibodies, LukS-PV was detected with a mouse monoclonal antibody (IBT Bioservices) and LukG expression was measured using a rabbit anti-LukB polyclonal Ab (IBT Bioservices). Specific reactivity to the target antigens and lack of cross-reactivity with other toxins was proven by immunoblotting using recombinant Hla, LukS-PV, LukF-PV, HlgA, HlgB, HlgC, LukE, LukD and LukGH. Antibody binding was detected using anti-human, rabbit or mouse HRP-conjugated goat F(ab’)_2_ fragments (Southern Biotech) with the enhanced chemiluminescence (ECL) immunoblotting detection reagent (GE Healthcare) at an ImageQuant LAS 4000 (GE Healthcare) Imaging station.

### Live-dead cell staining

PMNs (1 × 10^5^ cells/well) were exposed to *S. aureus* CS and mAbs in presence of 4 µM Calcein AM and 2 µM EthD-1 (LIVE/DEAD® Viability/Cytotoxicity Kit, Life Technologies) in 8-well µ-slides (Ibidi) in neutrophil medium. PMN survival was monitored by time-lapse microscopy using a Cell-R Live Imaging System (Olympus). Pictures were taken in 1 minute intervals for a total of 90 min and exported as movies with a 3 frames per second rate.

## Results

### Inactivation of the five leukocidins of S. aureus by two toxin neutralizing human mAbs, ASN-1 and ASN-2

**Figure 1.**
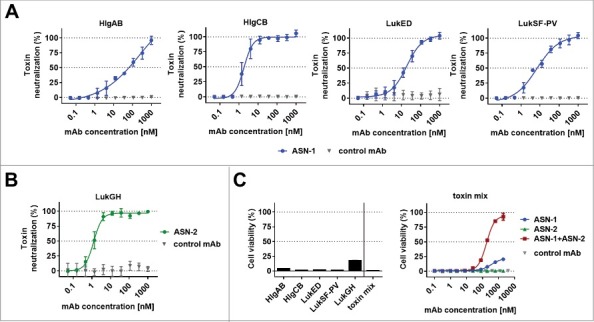
**Neutralization of recombinant leukocidins by ASN-1 and ASN-2. A**: Neutralization of recombinant HlgAB (10 nM), HlgCB (7.5 nM), LukED (10 nM) and LukSF-PV (5 nM) by ASN-1. **B**: Neutralization of LukGH (1 nM, TCH1516 LukGH) by ASN-2. **C**: left panel: Effect of individual toxins as in **A**, except for LukGH (1.875 nM each of the four LukGH variants expressed based on the MRSA252, MSHR1132, H19 and TCH1516 sequences) and a mixture thereof on PMN viability; right panel: Neutralization of a mixture of recombinant toxins by ASN-1, ASN-2 or ASN100 (1:1 mixture of ASN-1 and ASN-2). Antibody concentrations shown refer to individual mAbs. PMN viability was determined by luminescent quantification of cellular ATP levels. Data are derived from at least two independent experiments and shown as mean +/− SEM.

We measured leukocidin-mediated cytotoxicity towards polymorphonuclear cells (PMNs) in cell based viability assays by luminescent detection of ATP. ASN-1 preserved the viability of human PMNs exposed to recombinant HlgAB, HlgCB, LukED, and LukSF-PV (Fig. 1A), and ASN-2 fully neutralized recombinant LukGH (Fig. 1B).

Since *S. aureus* typically expresses several leukocidins, up to all five in some strains, antibody potency was also assessed in the presence of all five leukocidins. A mixture of recombinant toxins was used in cytotoxin neutralization assays at concentrations that reduced PMN viability by at least 80% (Fig. 1C). In these experiments, neither ASN-1 nor ASN-2 was able to inhibit toxicity alone; however, the equimolar combination of the two mAbs, called ASN100, was highly effective in blocking all five leukocidins and fully preserved cell viability.

### S. aureus isolates differ in their overall PMN toxicity and leukocidin expression profiles

**Figure 2.**
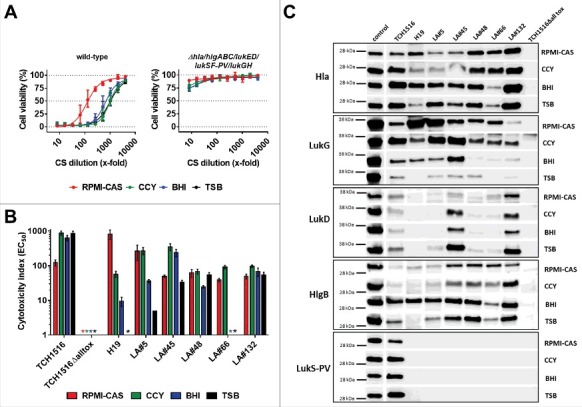
**Toxin expression profiles and neutrophil toxicity of *S. aureus* isolates. A**: Viability of human PMNs after exposure to serially diluted culture supernatants (CS) of USA300 CA-MRSA TCH1516 strain (left panel) and its isogenic *Δhla/hlgABC/lukED/lukSF-PV/lukGH* mutant (“TCH1516Δalltox“, right panel) grown in the indicated growth media. **B**: PMN toxicity of seven *S. aureus* strains expressed as CS dilution at the EC_50_. *) toxicity too low for EC_50_ calculation. PMN viability was determined by luminescent quantification of cellular ATP levels. Data are expressed as mean +/− SEM of three independent experiments. **C**: Immunoblot analysis of CS of seven *S. aureus* strains grown in the indicated media; control, recombinant toxin (0.1 µg/lane).

To characterize the effect of naturally-produced cytotoxins on neutrophils and to measure the effectiveness of the two toxin neutralizing antibodies, we exposed PMNs to *S. aureus* culture supernatants containing the secreted toxins. We used seven *S. aureus* isolates representing different genotypes: Four MRSA strains including a genome sequenced prototype USA300 CA-MRSA strain (TCH1516) and three MSSA strains with different sequence- (ST) and *spa*-types (Table 1). Five of the seven strains were collected from mechanically ventilated patients.^28^ To assess the contribution of the leukocidins to PMN toxicity in this assay, we generated a gene deletion mutant of the TCH1516 strain that lacked all five leukocidin genes (and additionally *hla*). Bacteria were grown to early stationary phase in four different growth media: three rich bacterial culture media, CCY, BHI, and TSB, most often used for culturing *S. aureus*, and RPMI, a mammalian cell culture medium (supplemented with casamino acids), to mimic *in vivo* conditions, such as low iron and nutrient content. The growth kinetics and optical densities after 8 hours growth when CSs were collected were comparable (Fig. S1). PMN toxicity was determined by serial dilution of the culture supernatants (CS) and expressed as the CS dilution that still resulted in a 50% reduction in cell viability (EC_50_) (example shown in Fig. 2A). Importantly, the CSs of the mutant strain lacking all six pore forming toxins did not display measureable EC_50_, and induced maximum 25% loss of viability at the lowest CS dilution. We also determined that Hla did not have an impact on viability of human PMNs in this assay (tested with recombinant Hla and CS of the TCH1516Δ*hlgABC/lukED/lukSF-PV/LukGH* mutant strain; data not shown). PMN toxicity in this *in vitro* model is therefore mainly driven by the leukocidins. Great differences were observed with the seven *S. aureus* strains in the same growth medium or with the same strain in different broths, with EC_50_ values ranging from 5 to approximately 900 (Fig. 2B). For example, while the LA#48 and LA#132 clinical isolates showed comparable toxicity when grown in the four different media, leukocidin expression of the livestock-associated MRSA strain H19 and the LA#66 isolate was greatly affected (>100-fold difference). Notably, CS generated with these two strains in TSB were not toxic to PMNs. All growth media considered, the *lukSF-PV+* USA300 CA-MRSA strain TCH1516 displayed the highest PMN toxicity.

Immunoblot analysis with cytotoxin specific antibodies revealed greatly variable expression levels of the individual leukocidins by the seven different *S. aureus* strains in the four different growth media tested (Fig. 2C), supporting the results of the PMN toxicity assays. LukG expression was detectable with all strains, with highest levels in RPMI-CAS and CCY, and low levels in TSB. HlgB levels were highest and comparable among strains in BHI medium, while three strains displayed lower levels in the other three media. Although *lukED* was present in six of the seven characterized isolates, only TCH1516, LA#45 and LA#132 showed detectable LukD expression in all four media. LukSF-PV expression was expected only with the TCH1516 strain based on the presence of *lukSF-PV* (Table 1). This toxin was detected in all four growth media, with slightly lower intensity in RPMI-CAS, compared to CCY, BHI and TSB. Since ASN-1 also binds Hla, which can compete for binding to the leukocidins, we also determined Hla levels in the bacterial CSs. Compared to the leukocidins, Hla levels were more uniform across the different culture supernatants, but still influenced by the growth media in a strain-dependent manner (Fig. 2C).

### Prevention of neutrophil lysis by S. aureus culture supernatants requires both ASN-1 and ASN-2

We measured neutralization of *S. aureus* CS in the presence of ASN-1, ASN-2 or both mAbs at 1 µM (150 µg/mL) concentration in several *in vitro* assays.

**Figure 3.**
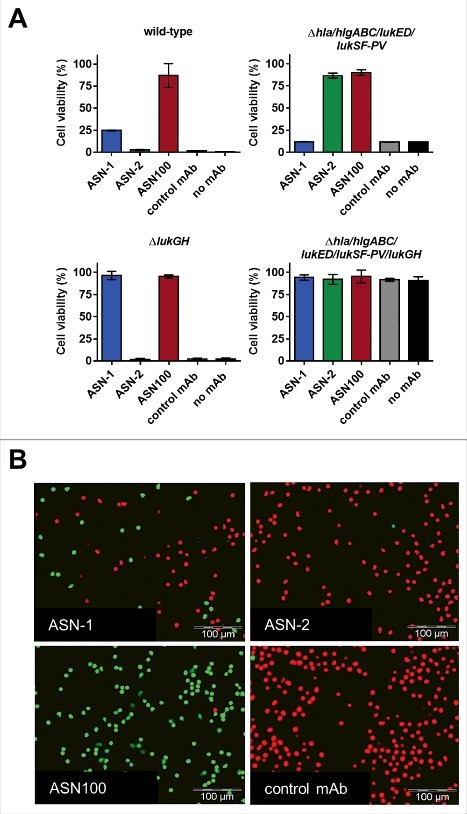
**Synergistic effect of ASN-1 and ASN-2 in neutralizing native *S. aureus* cytotoxins in bacterial culture supernatants (CS). A**: Human PMNs were exposed to CS of TCH1516 (at 8x dilution) and isogenic toxin gene deletion mutants grown in RPMI-CAS, in the presence of control antibody, ASN-1, ASN-2 or ASN100 (1 µM each). Data are shown as mean +/− SEM of two independent experiments. Viability was determined by luminescent quantification of cellular ATP levels. **B**: Calcein-AM/EthD-1 live/dead cell staining of human neutrophils after exposure to CS of TCH1516 (at 10x dilution) in the presence of ASN-1, ASN-2 and ASN100 (1 µM each) for 90 min. Live cells stained green, dead cells appear in red.

First, we utilized the ATP-based luminescent cell viability assay with human PMNs to assess the contribution of the individual mAbs to leukocidin neutralization. The TCH1516 strain grown in RPMI-CAS demonstrated almost 100% PMN cytotoxicity (using 8-fold diluted CS) in presence of an isotype control mAb (Fig. 3A, upper left panel). ASN-1 could partially inhibit toxicity while ASN-2 alone was ineffective. The equimolar mixture of the two mAbs, ASN100, preserved PMN viability (Fig. 3A, upper left panel). PMNs exposed to CS of the TCH1516*ΔlukGH* mutant were fully protected by ASN-1 (Fig. 3A, lower left panel), while those exposed to the CS of the TCH1516*Δhla/hlgABC/lukED/lukSF-PV* strain remained viable in the presence of ASN-2 (Fig. 3A, upper right panel). The CS of the TCH1516 strain lacking all toxin genes (TCH1516*Δhla/hlgABC/lukED/lukSF-PV/lukGH*) did not affect PMN viability (Fig. 3A, lower right panel).

Detection of pore formation by microscopy using Calcein-AM and EthD-1 for live/dead cell staining, mirrored the results obtained with the ATP-based luminescent assay (Fig. 3B). Time-lapse microscopy revealed that cell death in the control and ASN-2 containing samples occurred within minutes after exposure to TCH1516 CS. This effect was partially inhibited by ASN-1 and fully prevented in presence of both mAbs (Fig. 3B, Supplementary Movie File S1).

**Figure 4.**
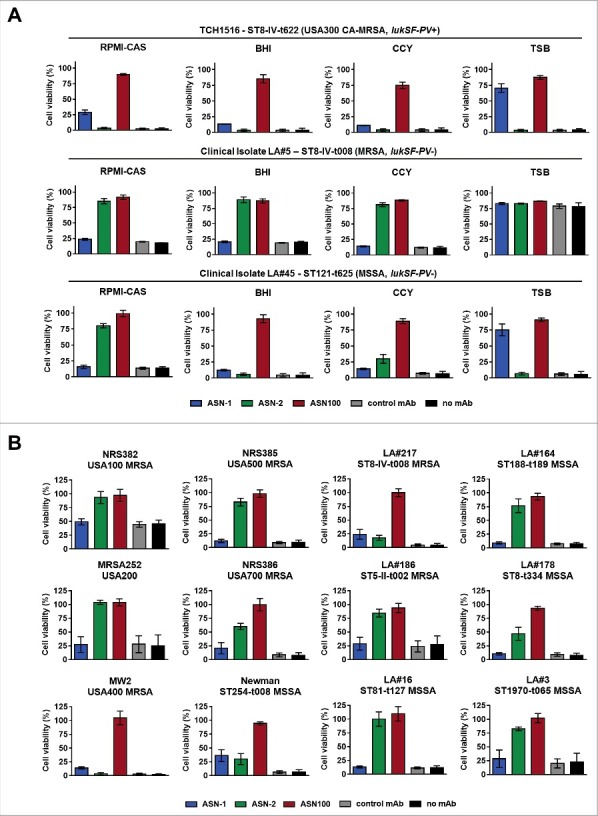
**Individual contributions of ASN-1 and ASN-2 to PMN protection are strain and growth condition dependent**. *S. aureus* isolates were grown in the indicated media, culture supernatants (at 16x dilution) were tested for PMN toxicity in the presence of ASN-1, ASN-2 and ASN100. **A**: Three representative isolates grown in different media. **B**: Six prototype strains and six clinical isolates grown in RPMI-CAS. PMN viability was determined by luminescent quantification of cellular ATP levels. MAbs were used at 1 µM each. Data are shown as mean +/− SEM of three independent experiments.

PMNs exposed to 28 *S. aureus* CS (seven strains grown in the four different media) were all protected by the equimolar mixture of ASN-1 and ASN-2 (ASN100), although greatly different patterns of protection were observed with the individual antibodies (Fig. 4A, Supplementary Fig. S2). Toxicity of the LukSF-PV-expressing USA300 strain was partially neutralized by ASN-1, but not at all by ASN-2, independent of the growth medium (Fig. 4A, upper panel). The CS of LA#5 (expressing high amounts of LukGH in RPMI, CCY and BHI medium) was neutralized with high potency by ASN-2 alone (Fig. 4A, middle panel). Neutralization of the LA#45 isolate displayed a mixed pattern dependent on the growth media (Fig. 4A, lower panel), which correlated well with the immunoblotting data (Fig. 2C). The high LukG production by this strain in RPMI medium (relative to LukD and HlgB) resulted in ASN-2 dominance, but a strong need for ASN-1 was observed in TSB medium (high LukD and HlgB levels, low LukGH). The BHI and CCY CSs contained comparable amounts of these three toxins, therefore, both mAbs were required for protection of PMNs (Fig. 4A, lower panel).

RPMI-CAS supernatants of a larger panel of *S. aureus* isolates, including the major MRSA pulsotypes USA100, USA200 (MRSA252), USA400 (MW2), USA500, and USA700, the Newman strain and 6 clinical isolates were also characterized (Table 1, Fig. 4B). Neutralization of PMN toxicity of *lukSF-PV*^+^ strains (TCH1516, LA#217, NRS386 and MW2) was generally dependent on both antibodies, while toxin activity mediated by *lukSF-PV*^−^ strains could be partially or fully inhibited by ASN-2 alone. Notably, we did not encounter a single *S. aureus* strain during these studies for which complete neutralization of toxicity was independent of ASN-2 in the low iron containing culture medium.

We also modeled the effect of different ASN-1 and ASN-2 ratios on PMN protection using CSs of various strains grown in different media. As the amount of any of the two mAbs was reduced in the neutralization assays (while one was kept constant), declining protection levels were observed with most supernatants (examples with TCH1516 and LA#132 strains grown in RPMI-CAS and CCY are shown in Fig. S3).

**Figure 5.**
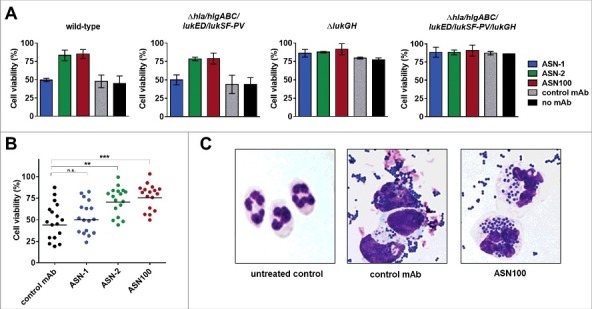
**Effect of ASN100 during PMN *ex vivo* infection**. Human PMNs were infected with different *S. aureus* strains at a MOI 50 for 2 hours. PMN viability was determined using a Calcein-AM viability dye. **A**: TCH1516 and isogenic mutant strains, mAbs used at 1 µM each. **B**: Data summary of TCH1516 and 16 additional clinical isolates, **p ≤ 0.01 and ***p ≤ 0.001 using one-way ANOVA with Dunn's multiple comparison test. Data are presented as mean +/− SEM of two independent experiments. **C**: May-Grünwald Giemsa staining of human PMNs 2 hours after infection with TCH1516, 100x magnification.

Since the presence of phagocytic cells upregulates leukocidin expression by *S. aureus*^40^ and PMN activating signals modulate cytotoxin susceptibility,^38^ we also assessed the relative contribution of ASN-1 and ASN-2 to preserving neutrophil viability in *ex vivo* infection experiments. PMNs were infected with different *S. aureus* strains at a starting multiplicity of infection (MOI) of approximately 50. PMN viability was determined after 2 hours with the fluorescent viability dye, Calcein-AM. The greatly reduced ability of the TCH1516Δ*lukGH* strain to kill PMNs, and the retained toxicity of the TCH1516Δ*hla*/*hlgABC/lukED/lukSF-PV* strain that was comparable to that induced by the wild-type strain, indicated a largely LukGH-dependent cytotoxic effect (Fig. 5A). In concordance with this, ASN-1 contributed little to the protection of PMNs from TCH1516, while ASN-2 alone was very efficient in maintaining viability comparable to ASN100. 16 other *S. aureus* strains (also tested in the culture supernatant mediated toxicity assay) were evaluated and found to induce 15% to 80% PMN cytotoxicity (Fig. 5B). In most cases, ASN-2 alone displayed protection levels comparable to ASN100, while ASN-1 alone was only marginally beneficial. These data suggest that under these experimental conditions, PMN death was largely mediated by LukGH with all *S. aureus* isolates tested.

Microscopic analysis of May-Grünwald-Giemsa stained PMNs exposed to TCH1516 live bacteria for two hours revealed that control samples (treated with isotype control mAb) showed swollen nuclei, altered cytoplasmic staining and few, if any, intracellular *S. aureus*, while in the presence of ASN100 cell morphology was preserved and neutrophils were able to engulf bacteria in large numbers (Fig. 5C). Notably, a similar outcome was achieved with the gene deletion mutant lacking the genes for all leukocidins and Hla in absence of neutralizing antibodies (Fig. S4).

### ASN100 prevents lysis of all human white blood cells susceptible to S. aureus leukocidins

**Figure 6.**
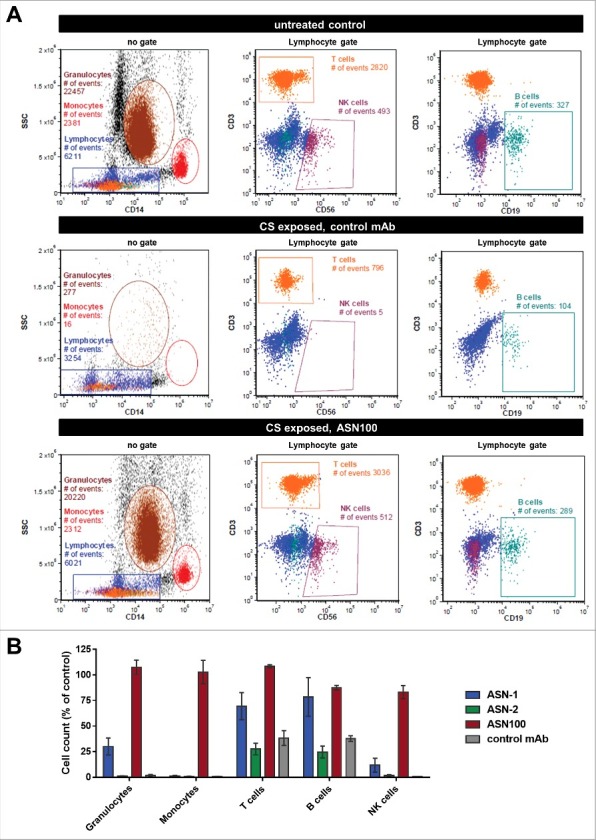
**Protection of white blood cells by ASN100**. Human peripheral WBCs were exposed to pooled BHI CS fractions of seven *S. aureus* strains for 2 hours and then stained with fluorescent antibodies specific for CD3, CD56, CD14, and CD19. Side-scatter and CD14 expression were used to discriminate lymphocyte, granulocyte and monocyte populations. T-cells, NK-cells and B-cells in the lymphocyte gate were further subtyped based on CD3, CD19 and CD56 expression. **A**: Representative plots showing untreated control cells and CS-exposed cells in presence of control mAb and ASN100. **B**: Data summary of 3 independent experiments using ASN-1, ASN-2 and ASN100. Data are shown as mean +/− SEM.

Although neutrophils are considered to be the major target cells of *S. aureus* leukocidins (all five are highly active on this cell type), lysis of other leukocyte types such as monocytes, lymphocytes and NK cells is also expected based on leukocidin receptor expression profiles.^11^ To assess the protective effect of ASN100 for these cell populations, we isolated peripheral white blood cells from fresh human blood and exposed them to CS pools of the seven strains characterized in Fig. 2. The different cell types were phenotyped by flow cytometry using side scatter properties in combination with cell surface markers (Fig. 6 and Fig. S5). Exposure to *S. aureus* CS almost completely eliminated granulocytes, monocytes, and NK-cells populations, and significantly reduced the number of T-lymphocytes and B-lymphocyte counts (Fig. 6). Similarly to the results obtained with purified PMNs in the viability assays, complete protection of granulocytes, monocytes, T-cells and NK cells was dependent on the simultaneous presence of both, ASN-1 and ASN-2 (Fig 6B). B-cell lysis was largely preventable with ASN-1 alone (Fig. 6B and Fig. S5). Importantly, ASN100 was effective in preserving the viability of all cell types analyzed.

### Protective effects of ASN100 in an *in vitro* 3D airway epithelial tissue model infected with S. aureus

**Figure 7.**
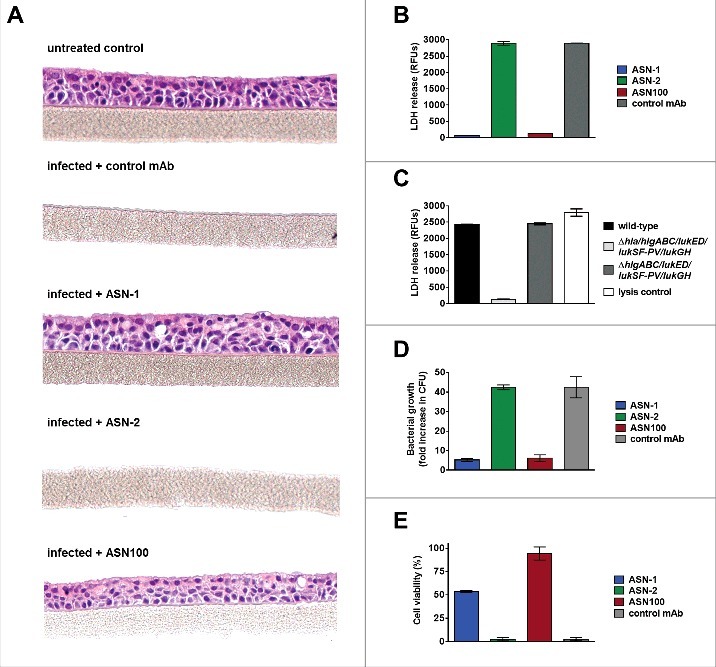
**ASN100 prevents Hla-mediated tissue damage in a human 3D lung tissue infection model**. EpiAirway™ tissues were infected with *S. aureus* TCH1516 at MOI 100 for 24 hours in the presence of control mAb, ASN-1, ASN-2 or ASN-100 (2 µM each). **A**: H&E staining of tissues. **B**: LDH release measured from basolateral compartment. **C**: LDH release measured with wild-type and gene deletion mutant TCH1516 strains in absence of mAbs. **D**: Relative increase in bacterial CFUs determined in the apical compartment. **E**: Viability of human neutrophils exposed to toxins produced in the control mAb sample in the presence of ASN-1, ASN-2 and ASN100 (1 µM each). Error bars indicate mean +/− SEM from duplicate measurements.

The cytoprotective effect of ASN-1 and ASN100 *via* Hla-neutralization was tested in a 3D- primary human respiratory epithelial tissues culture model (EpiAirway™, MatTek). These tissues consist of normal, human-derived tracheal/bronchial epithelial cells that are highly differentiated containing cilia and tight junctions and retain properties of normal respiratory tract epithelial tissue including active mucus secretion. Tissues were infected with the *S. aureus* TCH1516 strain using an MOI of 100 on the apical side for 24 hours in the presence of ASN-1, ASN-2, ASN100, or an isotype-matched control mAb. Hematoxylin-eosin staining revealed complete tissue destruction by *S. aureus* in samples with the control antibody and ASN-2, while ASN-1 and ASN100 treated samples displayed fully preserved tissue integrity (Fig. 7A). LDH release, measured in the basal feeding medium mirrored the microscopy data confirming full lysis of cells by *S. aureus* (comparable to saponin-treated samples), except when cells were co-incubated with ASN-1 or ASN100 (Fig. 7B). The lysis of epithelial cells was dependent on Hla, as the *hla*-deletion mutant *S. aureus* strain was not toxic, while the leukocidin gene deletion mutant strain was indistinguishable from the wild-type strain (Fig. 7C). Interestingly, we observed great reduction in bacterial counts of ASN-1 and ASN100 protected samples relative to control or ASN-2 treated samples (Fig. 7D). This is likely due to the growth enhancing effects of nutrients released from lysed cells, which is prevented in the presence of ASN-1.

Since no white blood cells are present in the MatTek EpiAirway™ model, Hla neutralization was sufficient to block *S. aureus* mediated toxicity. To indirectly assess leukocidin toxicity and ASN100 mediated protection of neutrophils, we collected the basolateral fluid of the tissues infected with *S. aureus* in the absence of toxin-neutralizing antibodies and tested for toxicity towards human neutrophils in presence of ASN-1, ASN-2 or ASN100. As bacteria cannot pass through the seeding membrane, only soluble molecules (such as toxins) reach the basolateral compartment. Performing neutralization assays with the harvested media containing the *in situ* produced toxins from the infected tissues we observed a similar pattern of PMN toxicity and ASN100 mediated protection as seen in bacterial culture media, namely partial neutralization with ASN-1, and full protection by ASN100 (Fig. 7E). This confirmed that *S. aureus* produced both Hla and multiple leukocidins during infection of the lung tissues and that all toxins could be neutralized by ASN100.

## Discussion

Given the high redundancy, species specificity, and variability of expression of *S. aureus* pore-forming cytotoxins, we applied a comprehensive approach to examine their effects on human target cells and to evaluate the potency of two human mAbs specific for alpha-hemolysin and five leukocidins in six different *in vitro* models. First, human PMNs, containing mainly neutrophils were exposed to the mixture of recombinant leukocidins. The two mAbs – ASN-1 that neutralizes four of the five leukocidins (in addition to Hla) and ASN-2 targeting the 5^th^ leukocidin, LukGH – were both required to prevent PMN cytotoxicity. Next, we characterized the leukocidin expression profiles of seven *S. aureus* isolates with different genetic backgrounds grown in four different culture media commonly used for *in vitro* culturing of *S. aureus*. Immunoblotting revealed variable expression levels of the individual leukocidins of the different strains that were influenced by the culture media. RPMI medium (optimized for mammalian cell cultures) is considered to be more appropriate to imitate *in vivo*-like conditions than rich bacterial culture media. Its low iron and nutrient content and physiologic ionic strength are expected to reflect the situation in plasma and tissue fluids. This assumption is supported by high promoter activity of the leukocidin genes in such media.^41^ The variable leukocidin expression in response to the different culture media was reflected by the PMN toxicity of the culture supernatants. Similar findings were made by quantitative mass-spectrometry and correlating individual leukocidin expression levels to the degree of PMN toxicity measured in RPMI versus rich culture medium.^42^

Cytotoxin expression is subject to complex regulation and influenced by environmental conditions. As seen with most secreted virulence factors, the cytotoxins are expressed mainly in the stationary growth phase *in vitro*. The global regulator Agr that is part of the quorum sensing (bacterium density) mechanism, is the master regulator of expression, but many other regulators, such as Sae, Rot, Sar, Rsp, and metabolic sensors have also been shown to modulate the expression of *S. aureus* cytotoxins.^43-46^ Mutations and/or variations in transcriptional regulators have been described in clinical *S. aureus* isolates. For example, a unique *saeS* allele was reported that overrides cell-density dependent SaeR and LukSF-PV expression in ST30 CA-MRSA isolates, resulting in a higher level of LukSF-PV in early log phase.^47^ Several studies report that *S. aureus* exposed to the host environment (human blood cells, infected animals in pneumonia model, surfactant, *etc.*) greatly up-regulates expression of *hla* and the leukocidin genes.^40,41,48-50^ Moreover, we found that exposure to inflammatory stimuli, such as LPS, *S. aureus* culture supernatant (peptidoglycan and delta-toxin), and IL-8, activates PMNs and alters their sensitivity towards the individual leukocidins, rendering LukGH the most dominant leukocidin *in vitro* due to up-regulation of its receptor, CD11b.^38^ Even without PMN stimulation, LukGH has been reported to be prominent among leukocidins in the culture supernatants of *S. aureus*.^42,51,52^

We found that ASN-1 or ASN-2 alone had variable effects on PMN viability upon exposure to the culture supernatants of 19 *S. aureus* strains, ranging from no effect to partial efficacy, and in some cases even full protection with ASN-2. Importantly, irrespective of the genetic background or culture medium used, the combination of the two mAbs, ASN100, was always sufficient to prevent PMN cytotoxicity. These data suggest that preventing immune cell damage necessitates the simultaneous neutralization of all leukocidins. *In vitro* infection of human PMNs with *S. aureus* revealed that LukGH was responsible for the majority of the killing effect and ASN-2 alone was highly protective. Microscopic analysis of PMNs protected by ASN100 detected intact cells that were able to engulf *S. aureus*; these intracellular bacteria were not seen in the controls. These data suggest that not only PMN viability but also phagocytic activity was maintained with ASN100.

Since leukocidins do not only target neutrophils, but also monocytes, macrophages and lymphocytes,^9,11^ we confirmed ASN100 efficacy and the requirement for both mAbs with these important cell populations.

Hla-mediated pathogenesis was mimicked by infecting primary human mucociliary tracheal/bronchial epithelial tissue cultured at the air-liquid phase with *S. aureus*. We observed full protection of epithelial cell morphology and prevention of LDH release by ASN-1 due to its Hla-neutralizing activity. Moreover, reduced growth of *S. aureus* in the presence of ASN-1 was also evident, most likely due to limited access to nutrients from lysed epithelial cells.

**Figure 8.**
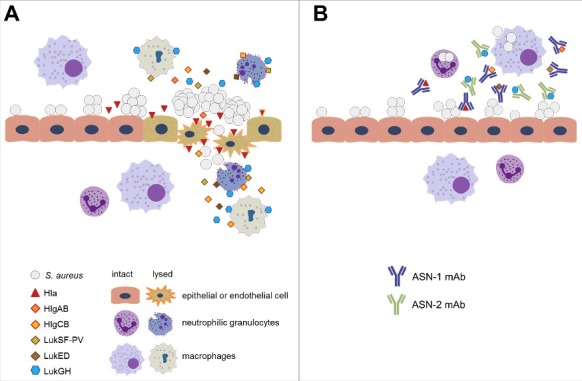
**Proposed model for the role of *S. aureus* cytotoxins in pathogenesis and mode of action of ASN100. A**: As bacterial density increases on mucosal surfaces, *S. aureus* secretes up to six cytolytic toxins. Alpha-hemolysin (Hla) lyses epithelial and endothelial cells allowing invasion to otherwise sterile body parts (e.g. lung, connective tissue) and promoting *S. aureus* disease (e.g. pneumonia, deep tissue infection). Up to five different leukocidins that primarily attack phagocytic cells, such as alveolar and tissue macrophages and granulocytes are also released, leading to weakened host defense and slower elimination of *S. aureus*. **B**: ASN-1 that neutralizes Hla and four leukocidins and ASN-2 that is specific for the fifth leukocidin (LukGH) inhibit cytotoxicity of *S. aureus*, which allows preservation of tissue integrity and immune defense during infection.

Based on the *in vitro* data presented here and the high *in vivo* efficacy in rabbit models,^15,54^ we propose that ASN100 treatment greatly reduces *S. aureus* virulence by preventing Hla-mediated tissue damage and subsequently bacterial invasion, and by preserving phagocyte function, enabling the human host to efficiently control bacterial growth (Fig. 8). In addition, prevention of neutrophil killing is expected to reduce inflammation and edema, especially in the lung, where recruited and subsequently lysed phagocytes damage the lung by releasing the contents of cytotoxic granules and/or reactive oxygen species.^17^ As antibiotics do not address these important virulence determinants, and are even implicated in enhancing cytotoxin production by *S. aureus*,^55^ adjunct therapy in addition to prophylaxis or preemptive applications is justified.

The limitation of our study is related to the lack of data on *in vivo* cytotoxin expression levels in patients. The increase in cytotoxin specific antibodies during convalescence from invasive *S. aureus* infections provide evidence that these virulence factors are indeed expressed in human *S. aureus* disease.^56,57^ Notably, the greatest increase in anti-cytotoxin antibody levels was seen with LukGH (LukAB).^57^

Although ASN-1 alone is able to neutralize five different cytotoxins, while ASN-2 can inhibit only one, the function of ASN-2 is crucial for the protection of phagocytic cells due to the dominant role of LukGH, especially under iron-restricted conditions and upon exposure of PMNs to inflammatory stimuli.^38^ Consistent with the importance of both mAbs and their synergistic mode of action, changing the ratios of ASN-1 or ASN-2 by decreasing one component and keeping the other constant was inferior in overall protective potency. Facing the highly variable leukocidin expression profiles across clinical isolates, our approach is to provide sufficiently high mAb levels in patients, not only in the plasma but also in epithelial lining fluids. In the ongoing Phase 2 clinical trial (NCT02940626), mechanically ventilated patients who become heavily colonized with *S. aureus* (based on microbiological analysis of ETA samples) receive equal amounts of ASN-1 and ASN-2 (1800 mg each). Based on the pharmacokinetic analysis from a healthy volunteer study, this ASN100 dose is expected to result in approximately 450 µg/mL initial and >150 µg/mL plasma levels of ASN-1 and ASN-2 for at least 2 weeks following intravenous administration.^58^

The recognition of the toxin-driven pathogenesis of *S. aureus* started almost 100 years ago and led to passive immunization trials in patients with complicated skin infections.^59,60^ Recent clinical studies with anti-*S.* *aureus* vaccines and passive immunization include or completely rely on anti-toxin approaches.^5,61^ These forthcoming studies will reveal whether inactivation of cytotoxins can result in prevention or amelioration of *S. aureus* infections in humans.

## Supplementary Material

KVIR_S_1391447.zip

## References

[1] Herrmann M, Smeltzer MS Clinical significance in humans In Staphylococcus: Genetics and Physiology. Somerville GA, editor United Kingdom (UK): Caister Academic Press, FL23-44;2016.

[2] Tong SYC, Davis JS, Eichenberger E, Holland TL, Fowler VG Staphylococcus aureus infections: epidemiology, pathophysiology, clinical manifestations, and management. Clin. Microbiol. Rev. 2015;28(3):603-61. doi:10.1128/CMR.00134-14 26016486 PMC4451395

[3] Spaan AN, Surewaard BG, Nijland R, van Strijp JA Neutrophils versus Staphylococcus aureus: a biological tug of war. Annu. Rev. Microbiol. 2014;67:629-50. doi:10.1146/annurev-micro-092412-155746 23834243

[4] Dryla A, Prustomersky S, Gelbmann D, Hanner M, Bettinger E, Kocsis B, Kustos T, Henics T, Meinke A, Nagy E Comparison of antibody repertoires against Staphylococcus aureus in healthy individuals and in acutely infected patients. Clin. Diagn. Lab. Immunol. 2005;12(3):387-98. 15753252 10.1128/CDLI.12.3.387-398.2005 PMC1065207

[5] Pozzi C, Olaniyi R, Liljeroos L, Galgani I, Rappuoli R, Bagnoli F Vaccines for Staphylococcus aureus and target populations. Curr Top Microbiol Immunol. 2016 Berlin, Heidelberg: Springer, 2016. doi: 10.1007/82_2016_54. 28197738

[6] Fowler VG Jr., Proctor RA Where does a Staphylococcus aureus vaccine stand? Clin. Microbiol. Infect. 2014;20(Suppl 5):66-75. doi:10.1111/1469-0691.12570 24476315 PMC4067250

[7] Alonzo F, Torres VJ The bicomponent pore-forming leucocidins of Staphylococcus aureus. Microbiol Mol Biol Rev. 2014;78(2):199-230. doi:10.1128/MMBR.00055-13 24847020 PMC4054254

[8] Vandenesch F, Lina G, Henry T Staphylococcus aureus hemolysins, bi-component leukocidins, and cytolytic peptides: a redundant arsenal of membrane-damaging virulence factors? Front. Cell. Infect. Microbiol. 2012;2:12. 10.3389/fcimb.2012.00012 PMC3417661 22919604

[9] Reyes-Robles T, Torres VJ Staphylococcus aureus pore-forming toxins. Curr Top Microbiol Immunol. Berlin, Heidelberg: Springer, 2016. doi:10.1007/82_2016_16 27406190

[10] Berube BJ, Bubeck Wardenburg J Staphylococcus aureus alpha-toxin: nearly a century of intrigue. Toxins (Basel) 2013;5(6):1140-66. doi:10.3390/toxins5061140 23888516 PMC3717774

[11] Spaan AN, van Strijp JAG, Torres VJ Leukocidins: staphylococcal bi-component pore-forming toxins find their receptors. Nat Rev Microbiol. 2017;15(7):435-447. doi:10.1038/nrmicro.2017.27 28420883 PMC5621924

[12] Anderson MJ, Anderson MJ, Lin YC, Gillman AN, Parks PJ, Schlievert PM, Peterson ML Alpha-toxin promotes Staphylococcus aureus mucosal biofilm formation. Front Cell Infect Microbiol. 2012;2:64. doi:10.3389/fcimb.2012.00064. 22919655 PMC3417397

[13] Scherr TD, Hanke ML, Huang O, James DB, Horswill AR, Bayles KW, Fey PD, Torres VJ, Kielian T Staphylococcus aureus biofilms induce macrophage dysfunction through Leukocidin AB and alpha-toxin. MBio. 2015;6(4):pii: e01021-15. doi:10.1128/mBio.01021-15 PMC4550693 26307164

[14] Alonzo F 3rd, Benson MA, Chen J, Novick RP, Shopsin B, Torres VJ Staphylococcus aureus leucocidin ED contributes to systemic infection by targeting neutrophils and promoting bacterial growth in vivo. Mol Microbiol. 2012;83(2):423-35. doi:10.1111/j.1365-2958.2011.07942.x 22142035 PMC3258504

[15] Diep BA, Le VT, Visram ZC, Rouha H, Stulik L, Dip EC, Nagy G, Nagy E Improved protection in a rabbit model of community-associated methicillin-resistant Staphylococcus aureus necrotizing pneumonia upon neutralization of leukocidins in addition to alpha-hemolysin. Antimicrob Agents Chemother. 2016;60(10):6333-40 27527081 10.1128/AAC.01213-16 PMC5038318

[16] Löffler B, Hussain M, Grundmeier M, Brück M, Holzinger D, Varga G, Roth J, Kahl BC, Proctor RA, Peters G Staphylococcus aureus Panton-Valentine leukocidin is a very potent cytotoxic factor for human neutrophils. PLoS Pathog. 2010;6(1):e1000715. doi:10.1371/journal.ppat.1000715 20072612 PMC2798753

[17] Diep BA, Chan L, Tattevin P, Kajikawa O, Martin TR, Basuino L, Mai TT, Marbach H, Braughton KR, Whitney AR, et al. Polymorphonuclear leukocytes mediate Staphylococcus aureus Panton-Valentine leukocidin-induced lung inflammation and injury. Proc Natl Acad Sci USA. 2010;107(12):5587-92. doi:10.1073/pnas.0912403107 20231457 PMC2851770

[18] Malachowa N, Kobayashi SD, Braughton KR, Whitney AR, Parnell MJ, Gardner DJ, Deleo FR Staphylococcus aureus leukotoxin GH promotes inflammation. J Infect Dis. 2012;206(8):1185-93. doi:10.1093/infdis/jis495 22872735 PMC3448972

[19] DuMont AL, Yoong P, Day CJ, 3rd Alonzo F, McDonald WH, Jennings MP, Torres VJ Staphylococcus aureus LukAB cytotoxin kills human neutrophils by targeting the CD11b subunit of the integrin Mac-1. Proc Natl Acad Sci U S A. 2013;110(26):10794-9. doi:10.1073/pnas.1305121110 23754403 PMC3696772

[20] Reyes-Robles T, Alonzo F 3rd, Kozhaya L, Lacy DB, Unutmaz D, Torres VJ Staphylococcus aureus leukotoxin ED targets the chemokine receptors CXCR1 and CXCR2 to kill leukocytes and promote infection. Cell Host Microbe. 2013;14(4):453-9. doi:10.1016/j.chom.2013.09.005 24139401 PMC3876884

[21] Alonzo F 3rd, Kozhaya L, Rawlings SA, Reyes-Robles T, DuMont AL, Myszka DG, Landau NR, Unutmaz D, Torres VJ CCR5 is a receptor for Staphylococcus aureus leukotoxin ED. Nature 2013;493(7430):51-5. 23235831 10.1038/nature11724 PMC3536884

[22] Spaan AN, Henry T, van Rooijen WJ, Perret M, Badiou C, Aerts PC, Kemmink J, de Haas CJ, van Kessel KP, Vandenesch F, Lina G, van Strijp JA The staphylococcal toxin Panton-Valentine leukocidin targets human C5a receptors. Cell Host Microbe. 2013;13(5):584-94. doi:10.1016/j.chom.2013.04.006. 23684309

[23] Spaan AN, Vrieling M, Wallet P, Badiou C, Reyes-Robles T, Ohneck EA, Benito Y, de Haas CJ, Day CJ, Jennings MP, et al. The staphylococcal toxins γ-haemolysin AB and CB differentially target phagocytes by employing specific chemokine receptors. Nat Commun. 2014;5:5438. doi:10.1038/ncomms6438 25384670 PMC4228697

[24] Spaan AN, Reyes-Robles T, Badiou C, Cochet S, Boguslawski KM, Yoong P, Day CJ, de Haas CJ, van Kessel KP, Vandenesch F et al. Staphylococcus aureus targets the Duffy antigen receptor for chemokines (DARC) to lyse erythrocytes. Cell Host Microbe. 2015;18(3):363-70. doi:10.1016/j.chom.2015.08.001 26320997 PMC4578157

[25] Wilke GA, Bubeck Wardenburg J Role of a disintegrin and metalloprotease 10 in Staphylococcus aureus alpha-hemolysin-mediated cellular injury. Proc Natl Acad Sci U S A. 2010;107(30):13473-8. doi:10.1073/pnas.1001815107 20624979 PMC2922128

[26] Gillet Y, Issartel B, Vanhems P, Fournet JC, Lina G, Bes M, Vandenesch F, Piémont Y, Brousse N, Floret D, Etienne J Association between Staphylococcus aureus strains carrying gene for Panton-Valentine leukocidin and highly lethal necrotising pneumonia in young immunocompetent patients. Lancet. 2002;359(9308):753-9. doi:10.1016/S0140-6736(02)07877-7 11888586

[27] del Giudice P, Blanc V, de Rougemont A, Bes M, Lina G, Hubiche T, Roudière L, Vandenesch F, Etienne J Primary skin abscesses are mainly caused by Panton-Valentine leukocidin-positive Staphylococcus aureus strains. Dermatology. 2009;219(4):299-302. doi:10.1159/000232391 19648730

[28] Stulik L, Malafa S, Hudcova J, Rouha H, Henics BZ, Craven DE, Sonnevend AM, Nagy E Hemolysin activity of methicillin-susceptible S. aureus predicts ventilator-associated pneumonia. Am J Respir Crit Care Med. 2014;190(10):1139-48. doi:10.1164/rccm.201406-1012OC 25303310

[29] Bubeck Wardenburg J, Schneewind O Vaccine protection against Staphylococcus aureus pneumonia. J Exp Med. 2008;205(2):287-94. doi:10.1084/jem.20072208 18268041 PMC2271014

[30] Foletti D, Strop P, Shaughnessy L, Hasa-Moreno A, Casas MG, Russell M, Bee C, Wu S, Pham A, Zeng Z, et al. Mechanism of action and in vivo efficacy of a human-derived antibody against Staphylococcus aureus alpha-hemolysin. J Mol Biol. 2013;425(10):1641-54. 23416200 10.1016/j.jmb.2013.02.008

[31] Hua L, Hilliard JJ, Shi Y, Tkaczyk C, Cheng LI, Yu X, Datta V, Ren S, Feng H, Zinsou R et al. Assessment of an anti-alpha-toxin monoclonal antibody for prevention and treatment of Staphylococcus aureus-induced pneumonia. Antimicrob Agents Chemother. 2014;58(2):1108-17. doi:10.1128/AAC.02190-13 24295977 PMC3910899

[32] Adhikari RP, Ajao AO, Aman MJ, Karauzum H, Sarwar J, Lydecker AD, Johnson JK, Nguyen C, Chen WH, Roghmann MC Lower antibody levels to Staphylococcus aureus exotoxins are associated with sepsis in hospitalized adults with invasive S. aureus infections. J Infect Dis. 2012;206(6):915-23. 22807524 10.1093/infdis/jis462

[33] Fritz SA, Tiemann KM, Hogan PG, Epplin EK, Rodriguez M, Al-Zubeidi DN, Bubeck Wardenburg J, Hunstad DA A serologic correlate of protective immunity against community-onset Staphylococcus aureus infection. Clin Infect Dis. 2013;56(11):1554-61. doi:10.1093/cid/cit123 23446627 PMC3641868

[34] Rasigade JP, Sicot N, Laurent F, Lina G, Vandenesch F, Etienne J A history of Panton–Valentine leukocidin (PVL)-associated infection protects against death in PVL-associated pneumonia. Vaccine. 2011;29(25):4185-6. doi:10.1016/j.vaccine.2011.04.033 21527300

[35] Rouha H, Badarau A, Visram ZC, Battles MB, Prinz B, Magyarics Z, Nagy G, Mirkina I, Stulik L, Zerbs M et al. Five birds, one stone: Neutralization of alpha-hemolysin and four bi-component leukocidins of Staphylococcus aureus with a single human monoclonal antibody. MAbs. 2015;7(1):243-54. doi:10.4161/19420862.2014.985132 25523282 PMC5045134

[36] Diep BA, Hilliard JJ, Le VT, Tkaczyk C, Le HN, Tran VG, Rao RL, Dip EC, Pereira-Franchi EP, Cha P et al. Targeting alpha toxin to mitigate its lethal toxicity in ferret and rabbit models of Staphylococcus aureus necrotizing pneumonia. Antimicrob Agents Chemother. 2017;61(4):pii: e02456-16. 10.1128/AAC.02456-16 PMC5365647 28115346

[37] Badarau A, Rouha H, Malafa S, Battles MB, Walker L, Nielson N, Dolezilkova I, Teubenbacher A, Banerjee S, Maierhofer B et al. Context matters: The importance of dimerization-induced conformation of the LukGH leukocidin of Staphylococcus aureus for the generation of neutralizing antibodies. MAbs. 2016;8(7):1347-60. doi:10.1128/AAC.02456-16. doi:10.1128/AAC.02456-16 27467113 PMC5058624

[38] Janesch P, Rouha H, Weber S, Malafa S, Gross K, Maierhofer B, Badarau A, Visram ZC, Stulik L, Nagy E Selective sensitization of human neutrophils to LukGH mediated cytotoxicity by Staphylococcus aureus and IL-8. J Infect. 2017;74(5):473-83. 28237625 10.1016/j.jinf.2017.02.004

[39] Badarau A, Rouha H, Malafa S, Logan DT, Håkansson M, Stulik L, Dolezilkova I, Teubenbacher A, Gross K, Maierhofer B et al. Structure-function analysis of heterodimer formation, oligomerization and receptor binding of the Staphylococcus aureus bi-component toxin LukGH. J. Biol. Chem. 2015;290(1): 142-56. doi:10.1016/j.jinf.2017.02.004. doi:10.1016/j.jinf.2017.02.004 25371205 PMC4281717

[40] Malachowa N, Whitney AR, Kobayashi SD, Sturdevant DE, Kennedy AD, Braughton KR, Shabb DW, Diep BA, Chambers HF, Otto M, DeLeo FR Global changes in Staphylococcus aureus gene expression in human blood. PLoS One. 2011;6(4):e18617. doi:10.1371/journal.pone.0018617 21525981 PMC3078114

[41] DuMont AL, Yoong P, Surewaard BG, Benson MA, Nijland R, van Strijp JA, Torres VJ Staphylococcus aureus elaborates the leukotoxin LukAB to mediate escape from within human neutrophils. Infect Immun. 2013;81(5):1830-41. 23509138 10.1128/IAI.00095-13 PMC3648020

[42] Chapman JR, Balasubramanian D, Tam K, Askenazi M, Copin R, Shopsin B, Torres VJ, Ueberheide BM Using quantitative spectrometry to understand the influence of genetics and nutritional perturbations on the virulence potential of Staphylococcus aureus. Mol Cell Proteomics. 2017;16(4 suppl 1):S15-S28. doi:10.1074/mcp.O116.065581 28196877 PMC5393389

[43] Bischoff M, Romby P Genetic regulation In Staphylococcus: Genetics and Physiology. Somerville GA, editor UK: Caister Academic Press, FL301-334;2016. doi:10.1128/IAI.00095-13

[44] Chua KY, Monk IR, Lin YH, Seemann T, Tuck KL, Porter JL, Stepnell J, Coombs GW, Davies JK, Stinear TP, Howden BP Hyperexpression of α-hemolysin explains enhanced virulence of sequence type 93 community-associated methicillin-resistant Staphylococcus aureus. BMC Microbiol. 2014;14:31. doi:10.1186/1471-2180-14-31 24512075 PMC3922988

[45] Li T, He L, Song Y, Villaruz AE, Joo HS, Liu Q, Zhu Y, Wang Y, Qin J, Otto M, Li M AraC-type regulator Rsp adapts Staphylococcus aureus gene expression to acute infection. Infect Immun. 2015;84(3):723-34. doi:10.1128/IAI.01088-15 26712209 PMC4771356

[46] Balasubramanian D, Ohneck EA, Chapman J, Weiss A, Kim MK, Reyes-Robles T, Zhong J, Shaw LN, Lun DS, Ueberheide B et al. Staphylococcus aureus coordinates leukocidin expression and pathogenesis by sensing metabolic fluxes via RpiRc. MBio. 2016;7(3):pii: e00818-16. doi:10.1128/mBio.00818-16 PMC4916384 27329753

[47] Ramundo MS, Beltrame CO, Botelho AM, Coelho LR, Silva-Carvalho MC, Ferreira-Carvalho BT, Nicolás MF, Guedes IA, Dardenne LE, O'Gara J, Figueiredo AM A unique SaeS allele overrides cell-density dependent expression of saeR and lukSF-PV in the ST30-SCCmecIV lineage of CA-MRSA. Int J Med Microbiol. 2016;306(6):367-80. 27265234 10.1016/j.ijmm.2016.05.001

[48] Palazzolo-Ballance AM, Reniere ML, Braughton KR, Sturdevant DE, Otto M, Kreiswirth BN, Skaar EP, DeLeo FR Neutrophil microbicides induce a pathogen survival response in community-associated methicillin-resistant Staphylococcus aureus. J Immunol. 2008;180(1):500-9. doi:10.1016/j.ijmm.2016.05.001. doi:10.1016/j.ijmm.2016.05.001 18097052

[49] Loughman JA, Fritz SA, Storch GA, Hunstad DA Virulence gene expression in human community-acquired Staphylococcus aureus infections. J Infect Dis. 2009;199(3):294-301. doi:10.1086/595982 19115951 PMC2843142

[50] Ishii K, Adachi T, Yasukawa J, Suzuki Y, Hamamoto H, Sekimizu K Induction of virulence gene expression in Staphylococcus aureus by pulmonary surfactant. Infect Immun. 2014;82(4):1500-10. doi:10.1128/IAI.01635-13 24452679 PMC3993393

[51] Ventura CL, Malachowa N, Hammer CH, Nardone GA, Robinson MA, Kobayashi SD, DeLeo FR Identification of a novel Staphylococcus aureus two-component leukotoxin using cell surface proteomics. PLoS One. 2010;5(7):e11634. doi:10.1371/journal.pone.0011634 20661294 PMC2905442

[52] Dumont AL, Nygaard TK, Watkins RL, Smith A, Kozhaya L, Kreiswirth BN, Shopsin B, Unutmaz D, Voyich JM, Torres VJ Characterization of a new cytotoxin that contributes to Staphylococcus aureus pathogenesis. Mol Microbiol. 2011;79(3):814-25. 21255120 10.1111/j.1365-2958.2010.07490.x PMC3312031

[53] Thammavongsa V, Kim HK, Missiakas D, Schneewind O Staphylococcal manipulation of host immune responses. Nat Rev Microbiol. 2015;13(9):529-43. doi:10.1038/nrmicro3521 26272408 PMC4625792

[54] Stulik L, Labrousse D, Croisier-Bertin D, Nagy G, Nagy E Efficacy of ASN100, a combination of two human monoclonal antibodies neutralizing Staphylococcus aureus cytotoxins in a CA-MRSA rabbit necrotizing pneumonia model. Poster presentation at European Congress of Clinical Microbiology and Infectious Diseases; Apr 22–25, Vienna, Austria.

[55] Stevens DL, Ma Y, Salmi DB, McIndoo E, Wallace RJ, Bryant AE Impact of antibiotics on expression of virulence-associated exotoxin genes in methicillin-sensitive and methicillin-resistant Staphylococcus aureus. J Infect Dis. 2007;195(2):202-11. doi:10.1086/510396 17191165

[56] Verkaik NJ, Dauwalder O, Antri K, Boubekri I, de Vogel CP, Badiou C, Bes M, Vandenesch F, Tazir M, Hooijkaas H, et al. Immunogenicity of toxins during Staphylococcus aureus infection. Clin Infect Dis. 2010;50(1):61-8. doi:10.1086/648673 19947854

[57] Thomsen IP, Dumont AL, James DB, Yoong P, Saville BR, Soper N, Torres VJ, Creech CB Children with invasive Staphylococcus aureus disease exhibit a potently neutralizing antibody response to the cytotoxin LukAB. Infect Immun. 2014;82(3):1234-42. doi:10.1128/IAI.01558-13 24379282 PMC3957992

[58] Magyarics Z, Jilma B, Leslie F, Luperchio S, Nagy E, Stevens C Safety and pharmacokinetics of ASN100, a monoclonal antibody combination for the prevention and treatment of Staphylococcus aureus infections, from a single ascending dose Phase 1 clinical study in healthy adult volunteers. Poster presentation at European Congress of Clinical Microbiology and Infectious Diseases; Apr 22–25, Vienna, Austria.

[59] Panton PN, Valentine FCO Staphylococcal toxin. Lancet 1932;219(5665):506-8. doi:10.1016/S0140-6736(01)24468-7

[60] Olaniyi R, Pozzi C, Grimaldi L, Bagnoli F Staphylococcus aureus-associated skin and soft tissue infections: Anatomical localization, epidemiology, therapy and potential prophylaxis. Curr Top Microbiol Immunol. Berlin, Heidelberg: Springer, 2016. doi: 10.1007/82_2016_32 27744506

[61] Sause WE, Buckley PT, Strohl WR, Lynch AS, Torres VJ Antibody-based biologics and their promise to combat Staphylococcus aureus infections. Trends Pharmacol Sci. 2016;37(3):231-41. doi:10.1016/j.tips.2015.11.008 26719219 PMC4764385

