# Supplementary material for: Disarming Staphylococcus aureus from destroying human cells by simultaneously neutralizing six cytotoxins with two human monoclonal antibodies
Source: Virulence. 2017 Dec 26;9(1):231–47. doi: 10.1080/21505594.2017.1391447 (PMC5955178; doi:10.1080/21505594.2017.1391447)
Supplement: KVIR_S_1391447.zip [file kvir-09-01-1391447-s001.zip › KVIR_S_1391447.docx]

**Online Supplementary Material.** The supplementary video shows time lapse microscopy of neutrophil survival in the presence of toxin neutralizing antibodies. Fig. S1 shows the growth curves of the *S. aureus* isolates characterized in Fig. 2. Fig. S2 demonstrates the synergistic effects of ASN-1 and ASN-2 against four *S. aureus* strains grown in four different culture media. Fig. S3 illustrates the effect of changing the ratio of ASN-1 and ASN-2 on PMN survival when exposed to bacterial culture supernatant. Fig. S4 depicts the effect of toxin gene deletion in *S. aureus* strain TCH1516 on phagocytosis by human neutrophils. Fig. S5 shows representative flow cytometry plots obtained with peripheral white blood cells when exposed to pooled *S. aureus* BHI CS fractions in presence of ASN-1 and ASN-2 alone.
