# Supplementary material for: Disarming Staphylococcus aureus from destroying human cells by simultaneously neutralizing six cytotoxins with two human monoclonal antibodies
Source: Virulence. 2017 Dec 26;9(1):231–47. doi: 10.1080/21505594.2017.1391447 (PMC5955178; doi:10.1080/21505594.2017.1391447)
Supplement: KVIR_S_1391447.zip [file kvir-09-01-1391447-s001.zip › KVIR_S_1391447_Figs.pptx]

## Slide 1
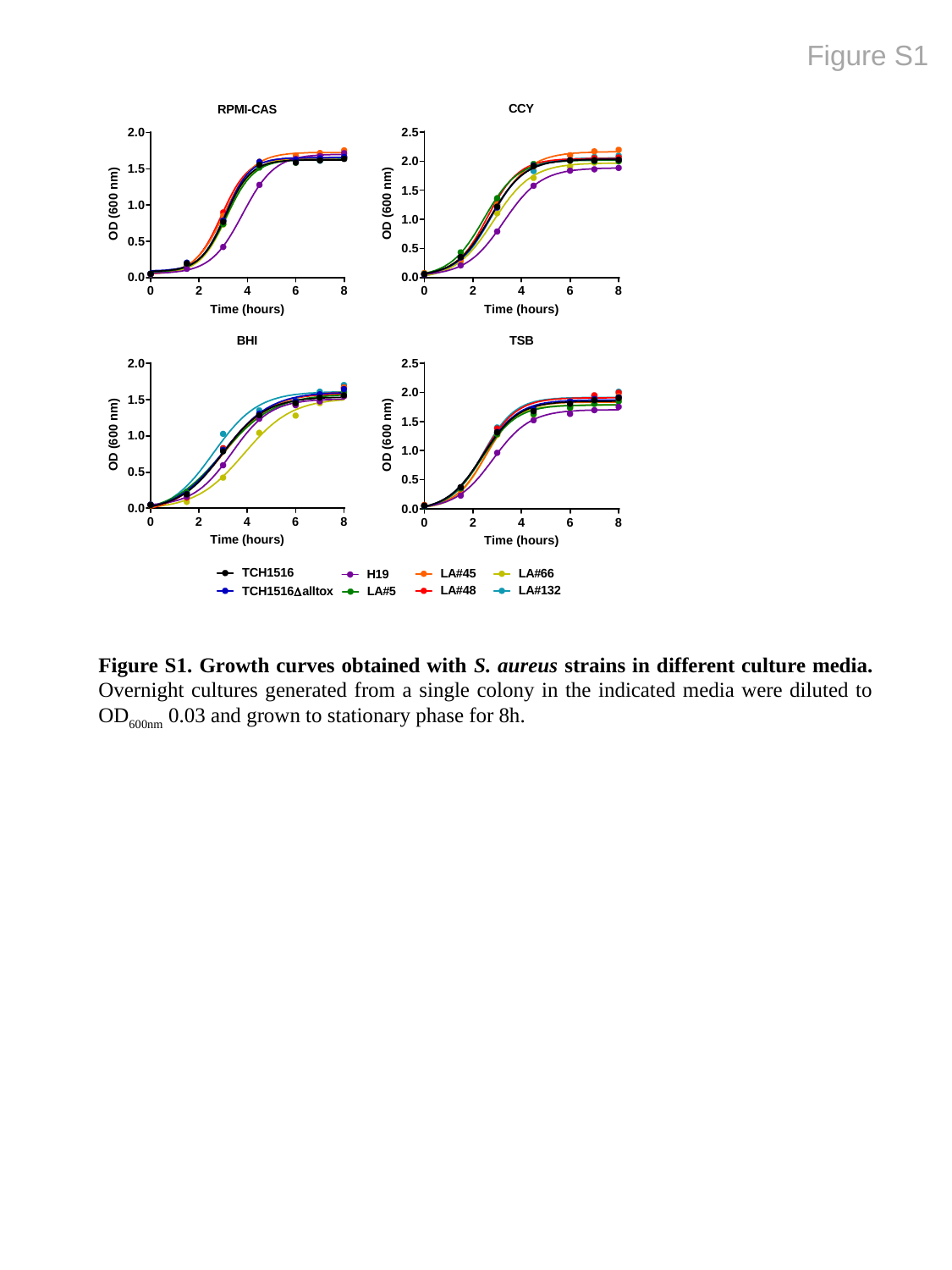

Figure S1
Figure S1. Growth curves obtained with S. aureus strains in different culture media. Overnight cultures generated from a single colony in the indicated media were diluted to OD600nm 0.03 and grown to stationary phase for 8h.

## Slide 2
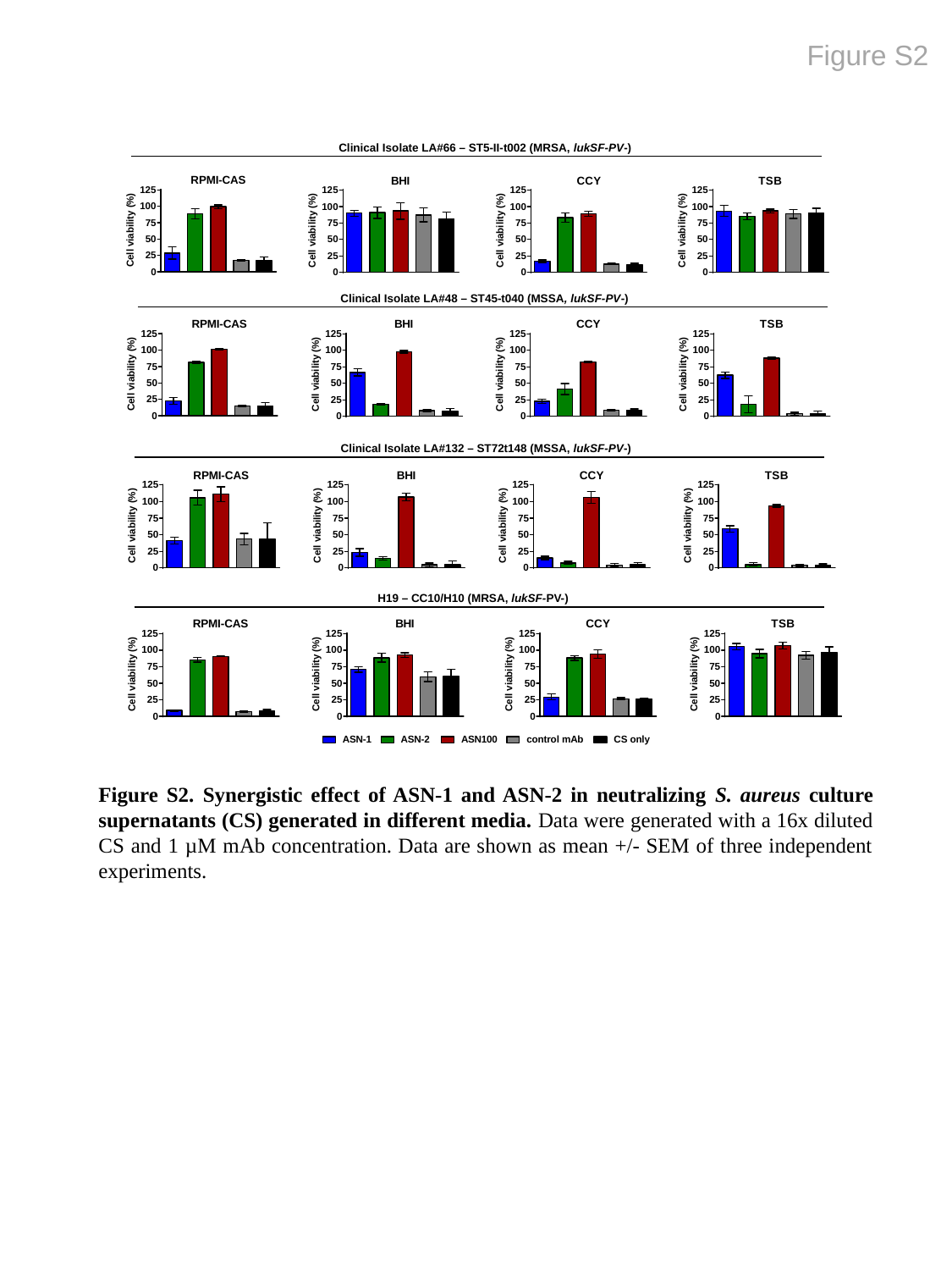

Figure S2
Clinical Isolate LA#66 – ST5-II-t002 (MRSA, lukSF-PV-)
Clinical Isolate LA#48 – ST45-t040 (MSSA, lukSF-PV-)
Clinical Isolate LA#132 – ST72t148 (MSSA, lukSF-PV-)
H19 – CC10/H10 (MRSA, lukSF-PV-)
Figure S2. Synergistic effect of ASN-1 and ASN-2 in neutralizing S. aureus culture supernatants (CS) generated in different media. Data were generated with a 16x diluted CS and 1 µM mAb concentration. Data are shown as mean +/- SEM of three independent experiments.

## Slide 3
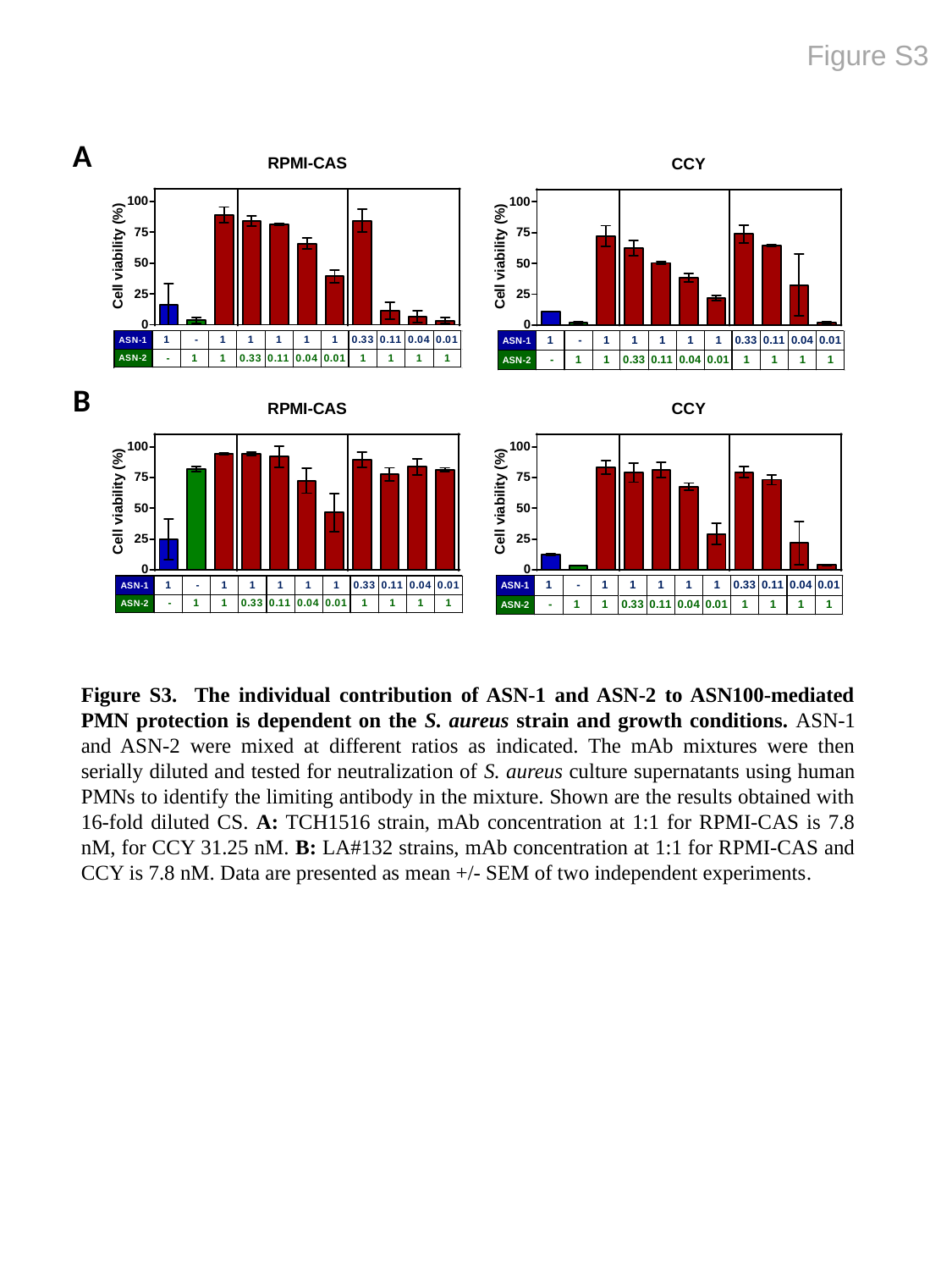

Figure S3
A
B
Figure S3. The individual contribution of ASN-1 and ASN-2 to ASN100-mediated PMN protection is dependent on the S. aureus strain and growth conditions. ASN-1 and ASN-2 were mixed at different ratios as indicated. The mAb mixtures were then serially diluted and tested for neutralization of S. aureus culture supernatants using human PMNs to identify the limiting antibody in the mixture. Shown are the results obtained with 16-fold diluted CS. A: TCH1516 strain, mAb concentration at 1:1 for RPMI-CAS is 7.8 nM, for CCY 31.25 nM. B: LA#132 strains, mAb concentration at 1:1 for RPMI-CAS and CCY is 7.8 nM. Data are presented as mean +/- SEM of two independent experiments.

## Slide 4
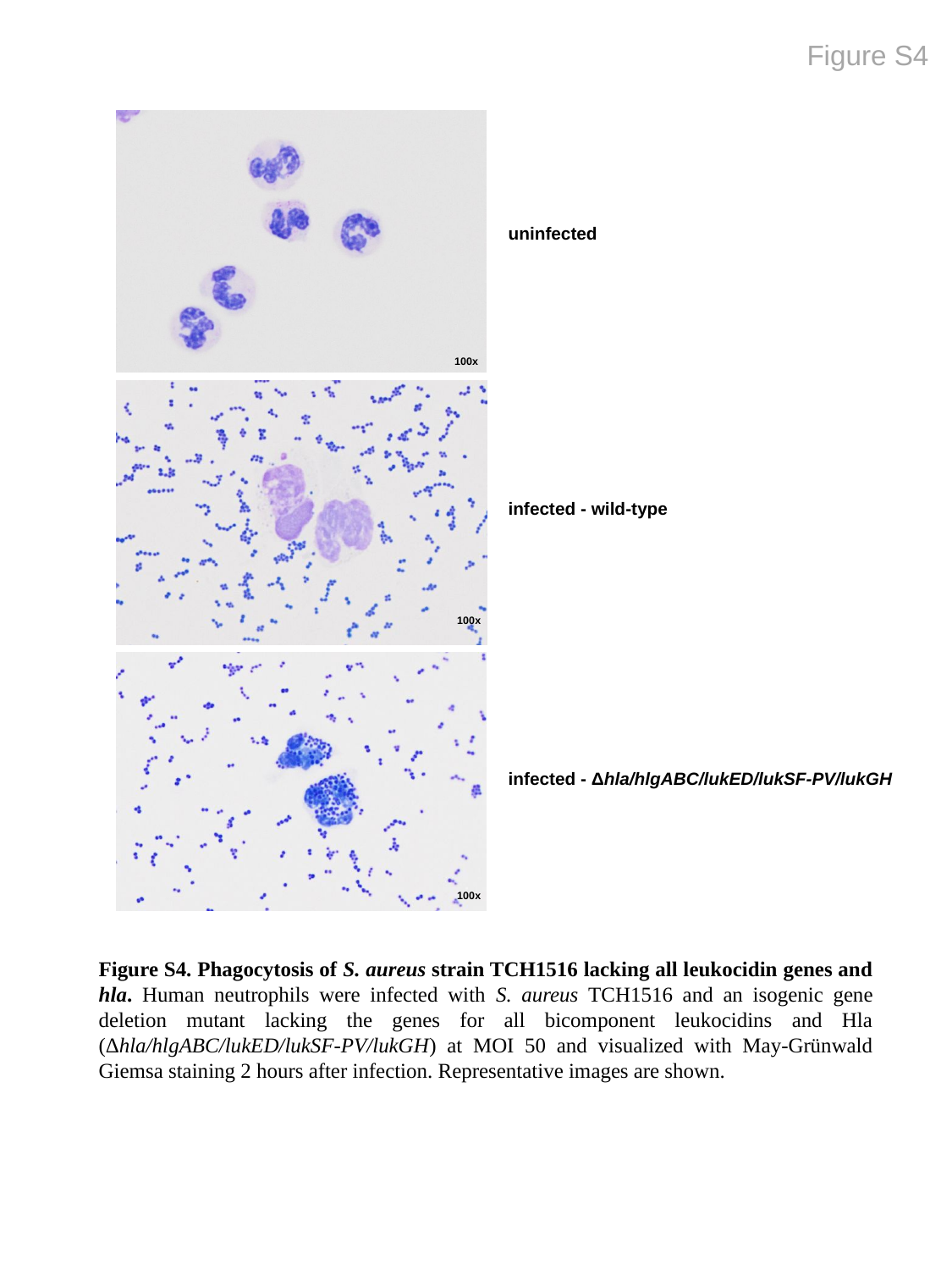

Figure S4
uninfected
100x
infected - wild-type
100x
infected - Δhla/hlgABC/lukED/lukSF-PV/lukGH
100x
Figure S4. Phagocytosis of S. aureus strain TCH1516 lacking all leukocidin genes and hla. Human neutrophils were infected with S. aureus TCH1516 and an isogenic gene deletion mutant lacking the genes for all bicomponent leukocidins and Hla (Δhla/hlgABC/lukED/lukSF-PV/lukGH) at MOI 50 and visualized with May-Grünwald Giemsa staining 2 hours after infection. Representative images are shown.

## Slide 5
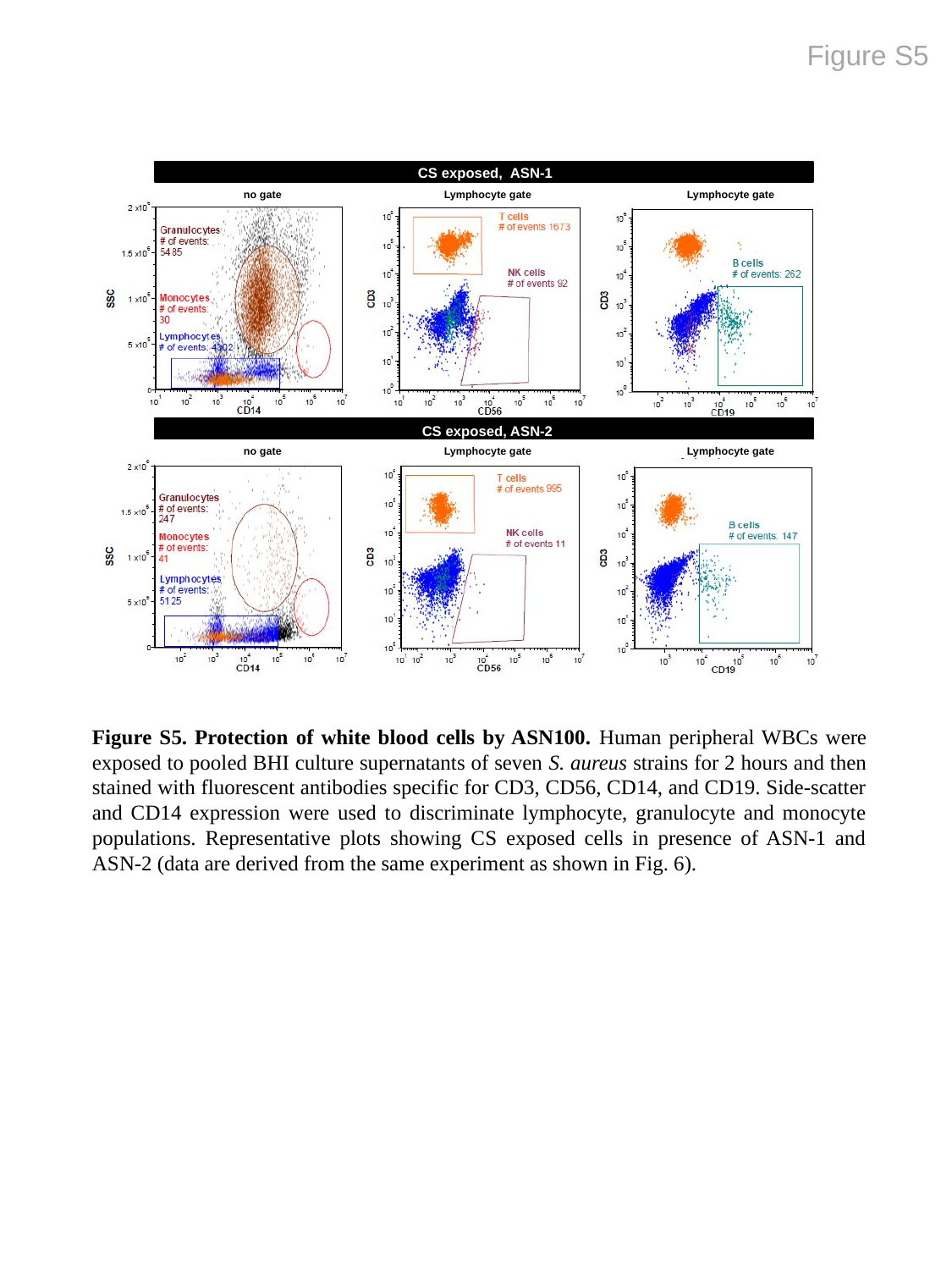

Figure S5
 CS exposed, ASN-1
no gate
 Lymphocyte gate
Lymphocyte gate
CS exposed, control mAb
 CS exposed, ASN-2
no gate
 Lymphocyte gate
Lymphocyte gate
Figure S5. Protection of white blood cells by ASN100. Human peripheral WBCs were exposed to pooled BHI culture supernatants of seven S. aureus strains for 2 hours and then stained with fluorescent antibodies specific for CD3, CD56, CD14, and CD19. Side-scatter and CD14 expression were used to discriminate lymphocyte, granulocyte and monocyte populations. Representative plots showing CS exposed cells in presence of ASN-1 and ASN-2 (data are derived from the same experiment as shown in Fig. 6).

## Slide 6
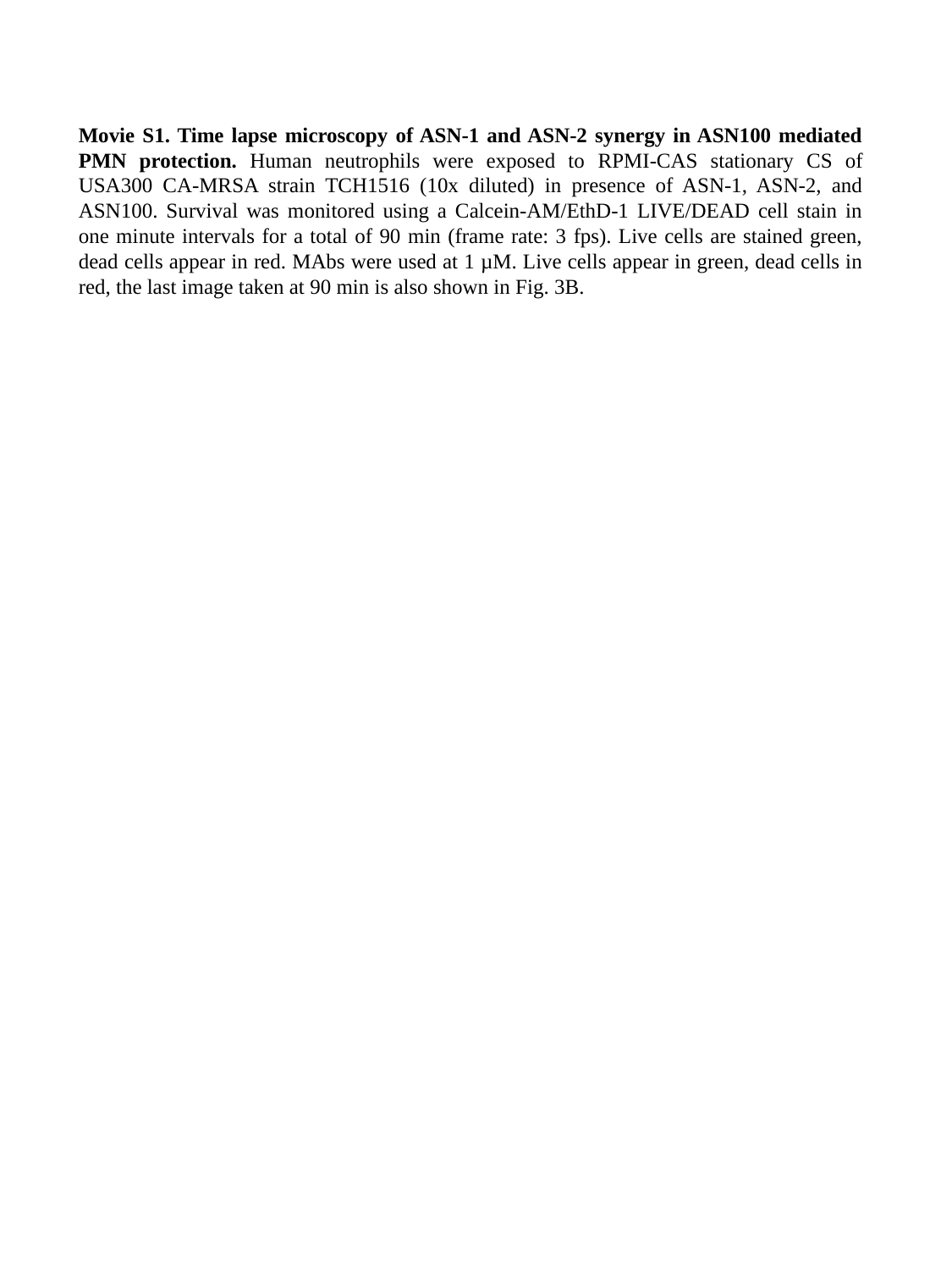

Movie S1. Time lapse microscopy of ASN-1 and ASN-2 synergy in ASN100 mediated PMN protection. Human neutrophils were exposed to RPMI-CAS stationary CS of USA300 CA-MRSA strain TCH1516 (10x diluted) in presence of ASN-1, ASN-2, and ASN100. Survival was monitored using a Calcein-AM/EthD-1 LIVE/DEAD cell stain in one minute intervals for a total of 90 min (frame rate: 3 fps). Live cells are stained green, dead cells appear in red. MAbs were used at 1 µM. Live cells appear in green, dead cells in red, the last image taken at 90 min is also shown in Fig. 3B.
